# Composition and Structural Design of Magnetic Alloy/Composites for High-Performance Microwave Absorption: A Review

**DOI:** 10.3390/nano16050290

**Published:** 2026-02-25

**Authors:** Mengyu Zhou, Zhuohui Zhou, Hongfei Cheng

**Affiliations:** Beijing Institute of Aeronautical Materials (BIAM), AECC, Beijing 100095, China; zhoumengyu0704@163.com (M.Z.);

**Keywords:** magnetic alloy, composite engineering, structural regulation, preparation technology, microwave-absorbing materials

## Abstract

Magnetic metals are of considerable importance for stealth technology and electromagnetic pollution control. However, they suffer from inherent limitations, such as the Snoek limit and narrow absorption bandwidth, which restrict their applications in complex scenarios. To address these challenges, this review systematically summarizes the recent advances of magnetic metal-based microwave-absorbing materials (MAMs), focusing on four core directions: alloy design, composite engineering, structural regulation, and preparation technology. The intensity and frequency bands of absorption in alloys are dictated by the material’s composition as well as its structural attributes. Moreover, composite systems incorporating carbon materials, MXenes, oxides, ceramics, and conductive polymers are discussed, where the synergistic design of components optimizes impedance matching and loss mechanisms. Key structural design strategies include core-shell structures, interface engineering, self-assembled hierarchical structures, and macroscopic structural design. These structures achieve the synergistic improvement of thin, lightweight, broadband, and strong absorption performance by enhancing interface polarization, multiple scattering, and resonance effects, while endowing materials with excellent environmental stability. Notably, metamaterial-based designs can further achieve an ultrawide bandwidth spanning 0.3–18 GHz. Additionally, preparation processes are crucial for regulating the microstructure and activating loss mechanisms. This review aims to offer theoretical and practical insights for developing high-performance, multifunctional magnetic MAMs.

## 1. Microwave Absorption Mechanisms

**Figure 1:**
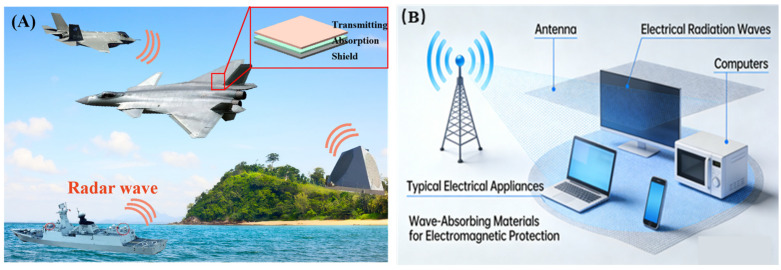
Typical application scenarios of (**A**) wave absorption and (**B**) electromagnetic protection.

MAMs have attracted considerable interest in recent years, driven primarily by growing demands in military stealth technology [1] (Figure 1A), 5G communication infrastructure deployment, electromagnetic compatibility and protection [2] (Figure 1B).

MAMs are generally categorized into two groups: dielectric loss types and magnetic loss types. Dielectric absorbers, particularly carbon-based materials, show excellent impedance matching at high frequencies but often encounter impedance mismatch in the low-frequency band (<2 GHz), which restricts their practical utility [3,4]. Among magnetic loss materials, magnetic metals typically possess higher permeability and saturation magnetization (M_s) in the GHz frequency range compared to ferrites [5], rendering them more suitable for low-frequency microwave absorption. Nevertheless, ferrites retain a distinct advantage in industrial applications due to their lower cost [6].

Fe, Co and Ni serve as representative magnetic metal absorbers [7,8,9], each offering distinct application advantages: Fe-based materials are preferred for low-frequency and cost-sensitive scenarios, Co-based materials perform exceptionally under high-frequency and high-temperature conditions, and Ni-based systems are valued for their room-temperature stability and processability. However, these materials commonly face challenges such as high density, skin effect, and agglomeration tendencies. In response, research on magnetic absorbers has evolved progressively, beginning with early stage simple alloying and material compounding, advancing to controlled synthesis of nanostructures, progressing further to microstructure design centered on heterogeneous interface regulation, and more recently moving toward multifunctional integration [10].

This review systematically summarizes recent advances in Fe-, Co- and Ni-based MAMs, with emphasis on alloy optimization, composite strategies, structural design, and preparation techniques. It aims to provide theoretical insights and technical guidance for the development of a new generation of microwave absorbers that integrate the desirable characteristics of thin thickness, wide bandwidth, and strong absorption.

### 1.1. Loss Mechanisms and Regulation Rules

Permeability can be described by the Globus equation [11]: $\mu_{i}=\frac{M_{s}^{2}}{{akH}_{c}M_{s}+b\lambda \xi }$, where λ and ξ are the magnetostriction coefficient and elastic strain parameter, respectively. Constants a and b are the material’s intrinsic composition, and k represents the proportional coefficient. This equation indicates that a higher static $M_{s}$ [A/m] and a lower coercivity ($H_{c}\;[A/m]$) are conducive to improving permeability. $M_{s}$ is predominantly governed by the chemical composition of the local atomic environment and the electronic structure, while $H_{c}$ mainly relies on the microstructure.

Magnetic loss primarily originates from Ref [12]: natural resonance, the eddy current effect, and exchange resonance within the GHz range. The natural ferromagnetic resonance frequency ($f_{r}$ [Hz]) is a key parameter determining the frequency adaptability of magnetic loss [13,14].

According to the Stoner–Wohlfarth theory [15],

(1) $${2\pi f}_{r}=\gamma H_{\alpha }$$

(2) $$H_{\alpha }=4|K|/\mu_{0}M_{s}$$

(3) $$K=\mu_{0}M_{s}H_{c}/2$$

γ, $H_{\alpha }$ [J] and $\mu_{0}$ are the gyromagnetic ratio, anisotropic energy and vacuum permeability ($4\pi \times 10^{-7}\;H/m$), respectively. K[J] is the magneto crystalline anisotropy constant. $f_{r}$ is mainly determined by K; the $f_{r}$ of bulk magnetic metals is generally in the order of MHz. Using element doping and stress anisotropy regulation, shape anisotropy design can shift ${\;f}_{r}$ of magnetic metals to the GHz range.

The frequency of exchange resonance is markedly higher than that of natural resonance. In a study by Rui Cai et al., the permeability spectra of their synthesized 1D Fe@Ni nanowires exhibited multiple distinct peaks across the 2–18 GHz band. Resonance peaks occurring in 2–8 GHz are attributed to exchange resonance, with the mechanisms shifting to natural resonance at frequencies exceeding 8 GHz [16].

Under electromagnetic excitation, induced eddy currents cause Joule heating and generate an opposing magnetic field that shields the interior from wave penetration, thereby reducing absorption efficiency. The expression for the eddy current loss contribution is given by the following:

(4) $$C_{0}=\mu^{\prime \prime }{\left(\mu^{\prime }\right)}^{-2}f^{-1}={2\pi \mu }_{0}\sigma d/3$$

**Figure 2:**
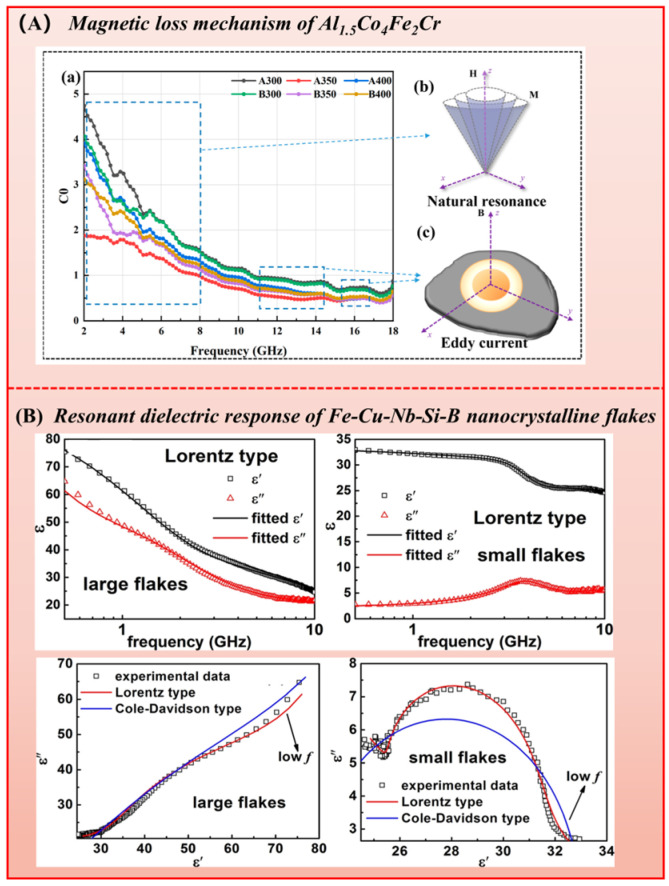
(**A**) Magnetic loss mechanism of Al_1.5_Co_4_Fe_2_Cr alloy [17], (**B**) Resonant dielectric response described by the Lorentz model of Fe-Cu-Nb-Si-B nanocrystalline flakes. (Reproduced with permission from Refs. [17,21]).

Based on this formula [16], if $C_{0}$ remains unchanged, the frequency dependence of eddy current loss can be neglected. Herein, σ [S/m], d [mm] correspond to electrical conductivity and material thickness, respectively. The primary strategy for mitigating this effect is achieved by designing nano, porous, or hollow structures, or through the application of insulating coatings [16]. It can be concluded that eddy current loss is prominent in Al_1.5_Co_4_Fe_2_Cr alloys [17], and their magnetic mechanism is illustrated in Figure 2A.

It should be noted that magnetic materials, particularly when formed into composites, also contribute to energy dissipation through dielectric loss. In the microwave regime, dielectric loss is dominated by interface polarization and dipole relaxation, with conduction loss being secondary. Polarization relaxation [18] is identified from peaks in the $\varepsilon^{\prime \prime }-f$ permittivity spectrum, which can be identified the Debye model [19]. The relaxation process of actual materials typically follows a multiple relaxation time distribution, which requires correction using the Cole–Cole model [20]. Its characteristic semicircles in the $\varepsilon^{\prime \prime }-\varepsilon^{\prime }$ plane represent individual Debye-type processes.

In addition to relaxation-type responses, resonant dielectric responses also exist in the microwave range. Originating from the forced resonance of polarized units in an alternating electric field, this phenomenon can be captured by the Lorentz model, which signature is a characteristic peak–valley structure in both $\varepsilon^{\prime }$ and $\varepsilon^{\prime \prime }$ near the resonance frequency. For instance, Yanhui Wu et al. [21] reported a strong such response between 0.5 and 10 GHz in composites with Fe-Cu-Nb-Si-B nanocrystalline flakes, as seen in Figure 2B.

### 1.2. Key Evaluation Metrics

The performance of an ideal MAM is governed by two synergistic requirements: First, excellent impedance matching ($|Z_{in}/Z_{0}|\approx 1$) to maximize wave entry with minimal reflection. Second, a substantial attenuation constant (α ≫ 1) to guarantee rapid dissipation of the incident electromagnetic energy.

For an MAM backed by a perfect conductor under normal wave incidence, the absorption performance is quantified by the reflection loss (RL [dB]), calculated as follows [22]:

(5) $$Z_{in}=Z_{0}\sqrt{\frac{\mu }{\varepsilon }}tanh(j\frac{2\pi fd}{c}\sqrt{\mu \varepsilon })$$

(6) $$RL=20log|\frac{Z_{in}-Z_{0}}{Z_{in}+Z_{0}}|$$

where $Z_{0}[\Omega ]=\sqrt{\mu_{0}/\varepsilon_{0}}$ is the free-space impedance, c is the speed of light, d [mm] is the material thickness, and ε and μ are the relative complex permittivity and permeability of the MAM, respectively.

The performance of MAMs is benchmarked by two key metrics: the minimum reflection loss (${RL}_{min}$) and the effective absorption bandwidth (EAB) [22]. The EAB specifically denotes the frequency band for which the RL reaches or exceeds −10 dB, a threshold indicating 90% microwave absorption.

Furthermore, the absorption frequency can be rationally designed by applying the quarter-wavelength matching principle. Optimal cancelation occurs at a specific frequency where the material thickness induces a half-wavelength path difference [23]

(7) $$t_{m}[\mathrm{mm}]=n\lambda /4=nc/4f_{m}\sqrt{\varepsilon_{r}\mu_{r}}$$

where $f_{m}$ [Hz] is the matching frequency; electromagnetic waves interfere destructively with the reflected waves from the conductive plate inside the material, achieving the ${RL}_{min}$.

## 2. Alloy Design

Alloying is a critical strategy to overcome the performance bottlenecks of single-metal MAMs and achieve customizable regulation of electromagnetic parameters [24].

### 2.1. Compositional Tuning

Composition plays a critical role in tailoring the electromagnetic properties of alloy absorbers [25,26]. Alloy compositions modify the total magnetic moment, govern the magnetic exchange interactions, and can trigger crystalline phase transitions, thereby altering the alloy’s electrical properties. Specific compositions such as Fe_20_Ni_80_ [27] and Fe_7_Co_3_ [28] exhibit broadband absorption. In FeSiAl systems, specific Si and Al contents (e.g., 9.6% Si and 5.4% Al) promote the formation of a D0_3_-type ordered superlattice, which markedly reduces $K_{1}$ and $\lambda_{s}$, resulting in very high initial permeability [29]. Heusler-type NiMnGa alloys show high sensitivity of Curie temperature, phase transformation, and magnetization to stoichiometry, with Ni_2_MnGa delivering strong wideband absorption from 6.3 to 18.0 GHz [30].

High-entropy alloys (HEAs) further extend this compositional strategy, utilizing high-configurational entropy and lattice distortion to disrupt conventional alloy paradigms. Systems such as FeCoNiCrAl [31], FeCoNiCrMn [32], and FeCoNiCuC_0.37_ [33] enable tailored phase and impedance characteristics through elemental adjustment.

Strategic doping can also effectively modulate their microwave absorption properties. In FeSiAl systems, the introduction of Cr induces magnetoelastic anisotropy at the interfaces between the D0_3_ and α-Fe phases [34]. Gd doping promotes the formation of heterogeneous interfaces [35]. The incorporation of a small amount of B is effective in refining the grain structure and enhancing mechanical properties [36]. The incorporation of Ti increases electrical resistivity, suppresses eddy current losses, and extends the effective absorption bandwidth. Flaky nanocrystalline FeTiSiAl powders with an aspect ratio of 25:1 exhibit excellent absorption performance from 100 MHz to 2.65 GHz [37].

From binary alloys to high-entropy and doping systems, precise compositional control is essential for breaking through the performance boundaries of alloy-based absorbers.

### 2.2. Crystallization Control

The crystallization characteristics of materials (e.g., crystal phase, grain size, and crystallinity) act as a critical bridge connecting their composition and macroscopic microwave absorption performance. The crystal phase structure determines the intrinsic magnetic properties (e.g., $K_{1}$ and $M_{s}$). For example, the FeSiAl alloy exhibits a progressive phase evolution, following the sequence of A2→A2/B2→A2/B2/D0_3_ during annealing [38]. This progression significantly reduces $H_{c}$, releases magnetic potential, and enhances permeability.

While grain size exerts a significant influence on magnetic loss mechanisms, when refined below the single-domain threshold (~40 nm for FeCo alloys), magnetic loss shifts toward more effective natural and exchange resonances in the GHz range. Nanocrystalline FeSiBPCu with grain sizes refined to 6 nm maintains high $M_{s}$ (182.3 emu/g) while achieving strong absorption (−44.0 dB) and an EAB of 9.2–15.0 GHz [39]. Enhanced crystallinity and structural ordering generally improve magnetic properties, though often at the cost of increased eddy current and reduced polarization-related losses, revealing a trade-off in loss behavior.

In this context, amorphous/nanocrystalline composite structures offer a balanced solution: the amorphous matrix suppresses eddy currents via high resistivity, while embedded nanocrystals maintain high saturation magnetization. In FeSiCr systems [40], increasing Cr content promotes amorphization, lowers permittivity, and optimizes impedance matching, achieving effective broadband absorption at 8 at% Cr. This synergy enables coordinated regulation of electromagnetic parameters, overcoming the performance limitations of single-phase structures.

### 2.3. Morphology and Structure

Structural innovation represents a pivotal strategy for enhancing microwave absorption performance. Comparative studies between granular and flaky FeNi alloys [41] reveal that flaky structures enhance permittivity through improved electrical conductivity and specific surface area, which jointly promote space-charge and interfacial polarization. Theoretical analyses based on the Landau–Lifshitz–Gilbert equation [42] indicate that flaky particles possess a higher magnetic anisotropy field, leading to a larger product of $M_{s}$ and $f_{r}$, which favors high complex permeability. Moreover, increasing the aspect ratio suppresses eddy current loss, further optimizing high-frequency magnetic responses.

The Landau–Lifshitz–Gilbert equation is expressed as follows:

(8) $$(\mu_{s}-1)f_{r}=\frac{\gamma M_{s}}{2\Pi }\sqrt{\frac{H_{ha}}{H_{ea}}}$$

The parameters $H_{ha}$ [A/m] and $H_{ea}$ [A/m] quantify the effective anisotropy fields generated by deviations of the magnetization from the easy axis within the hard and easy planes, respectively.

Another prominent example is the flattening treatment of FeSiAl particles, a critical process for optimizing their electromagnetic properties in the P–L bands [43]. Zhang et al. [44] demonstrated that increasing the aspect ratio of FeSiAl powders induces a redshift of the absorption peak toward lower frequencies. At a matching thickness of 6.5 mm, the flaky FeSiAl alloy achieves a reflection loss of −16.89 dB at 1 GHz.

3D hierarchical, such as flower-like [45] and dendritic morphologies [46], and Janus architectures [47] further extend loss mechanisms through enlarged surface areas and asymmetric interfaces. Additionally, core-shell designs [16,48], including core-air-shell configurations [49], optimize impedance matching and interfacial polarization. These structural paradigms collectively enable superior control over electromagnetic properties, yielding materials with strong, wideband absorption at reduced thicknesses.

### 2.4. Preparation and Post-Treatment Processes

The processing techniques, especially the final post-treatment, dictate the microstructure, thereby controlling the microwave absorption capabilities of magnetic alloys. Liquid-phase reduction [50] and mechanical alloying [51] enable precise control over composition and particle size, though challenges remain in scalability and purity. Optimized processes such as surfactant-assisted ball milling can refine particle morphology and suppress eddy current losses. For instance, Yining Li introduced ethanol as a process control agent, producing FeSiAl flakes with smooth surfaces and rounded edges [52]. This morphological optimization effectively enhances the permeability and microwave absorption loss properties in the 0.3–2 GHz range.

**Figure 3:**
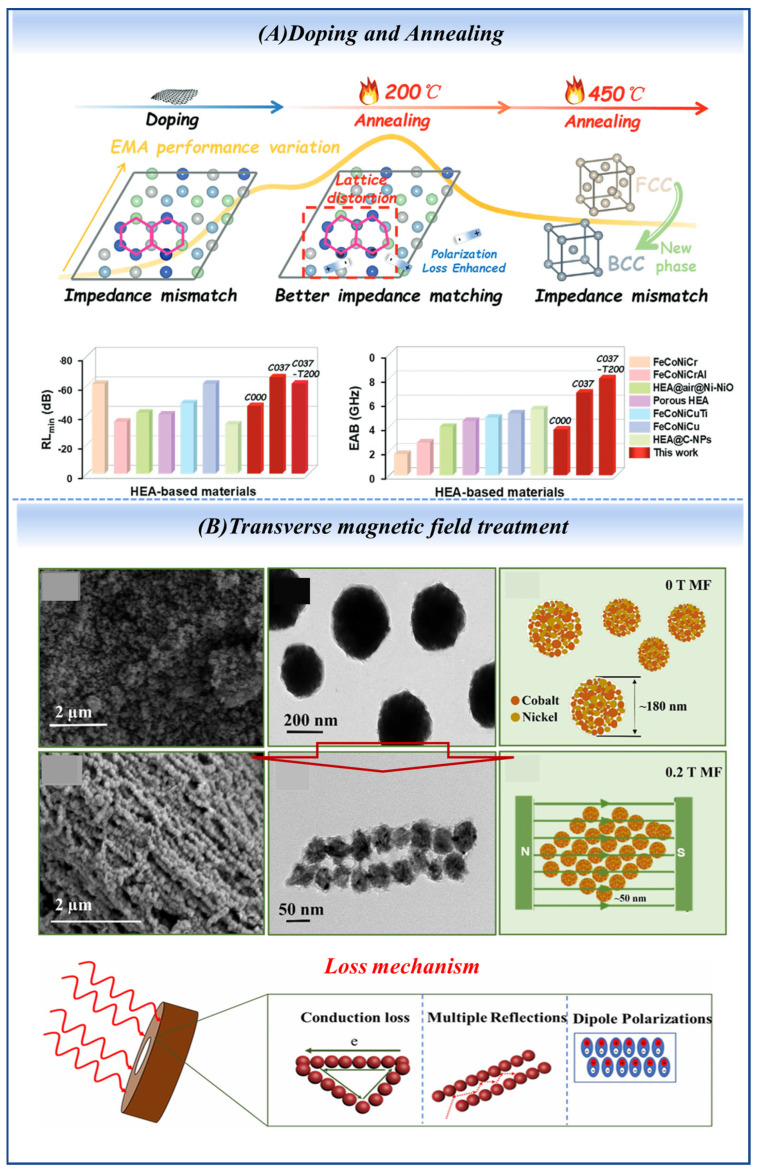
The post-treatment strategies include (**A**) annealing and (**B**) static magnetic field treatment. (Reproduced with permission from Refs. [33,58]).

Heat treatment [38] modulates crystallization behavior and phase composition to tailor electromagnetic properties, yet inappropriate temperatures may induce phase transformation and performance degradation. FeCoNiCuC_0.37_ [33] has an EAB of 7.99 GHz at 1.95 mm thickness after annealing at 200 °C. However, when annealing temperature exceeds 400 °C, its crystal structure transforms from FCC to BCC phase, and impedance matching deteriorates. The electronic structures and changes in crystal forms of FeCoNiCuC_0.37_ are depicted in Figure 3A. In Pr_2_Fe_17−x_Ni_x_ alloys, annealing at 100 °C for 2 h significantly improves absorption, with Pr_2_Fe_16_Ni reaching −23.6 dB at 2.72 GHz. This stems from internal stress relief and the formation of soft magnetic phases [53]. Similarly, step aging (500 °C, 10 h) of Fe–25Cr–12Co alloy allows tuning of the permeability peak within 0.3–3.3 GHz, enabling −20 dB absorption at 1.6 GHz [54].

Surface treatments effectively adjust permittivity and enhance environmental stability. Phosphating of flaky FeSiAl [55] effectively suppresses electron migration and reduces permittivity. The introduction of sodium stearate as a surfactant during FeCo particle synthesis progressively decreases permittivity. When modified with 0.004 mol of sodium stearate, the composite yields an EAB of 2.2 GHz in the C-band for a 2.7 mm thickness [56]. Additionally, surface treatments such as nitridation or oxidation [57] effectively enhance the environmental stability of absorbing materials.

As an emerging technique, magnetic field treatment can induce self-assembled chain-like structures and optimize magnetic loss mechanisms. Under a static field, CoNi nanospheres [58] self-assemble into chain-like structures (Figure 3B). Hui Zhao et al. [59] applied a transverse magnetic field of 200 kA/m to Fe_50_Ni_50_ alloy powders. This treatment significantly enhances the complex permeability over the 1–16 GHz range, with the improvement primarily attributed to internal stress release and magnetic structure optimization.

**Figure 4:**
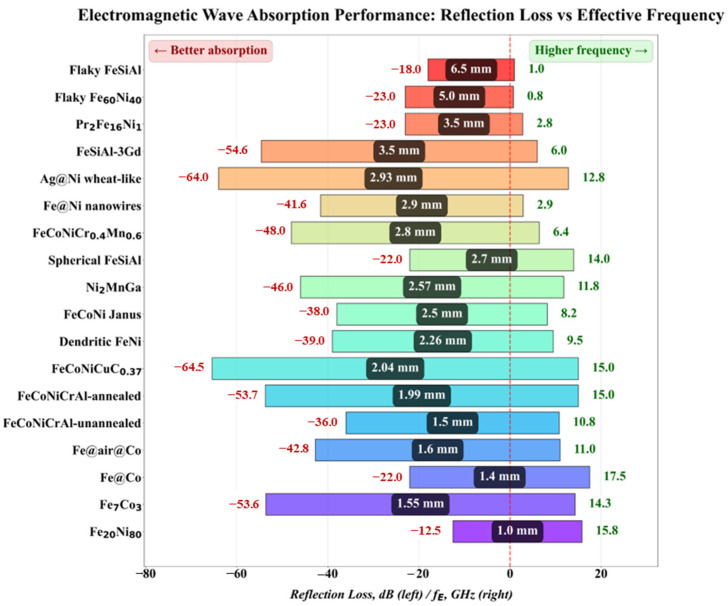
${RL}_{min}$ and corresponding frequency points at matching thicknesses of typical alloy systems (The data bars from top to bottom are derived from Refs. [42], [41], [54], [35], [48], [16], [32], [42], [30], [47], [46], [33], [31], [31], [49], [49], [28] and [27], respectively).

In summary, the rational selection of synthesis methods combined with synergistic post-treatment processes allows precise control over microstructure, phase composition, and interface characteristics, providing a robust technical foundation for designing high-performance MAMs. The preparation methods, mass ratios, EAB, ${RL}_{min}$, corresponding frequency points, and matching thicknesses of representative alloy systems are listed in Table 1 below. Furthermore, typical alloy systems have been selected to graphically illustrate their ${RL}_{min}$ and corresponding frequency points at matching thicknesses, as presented in Figure 4.

**Figure 5:**
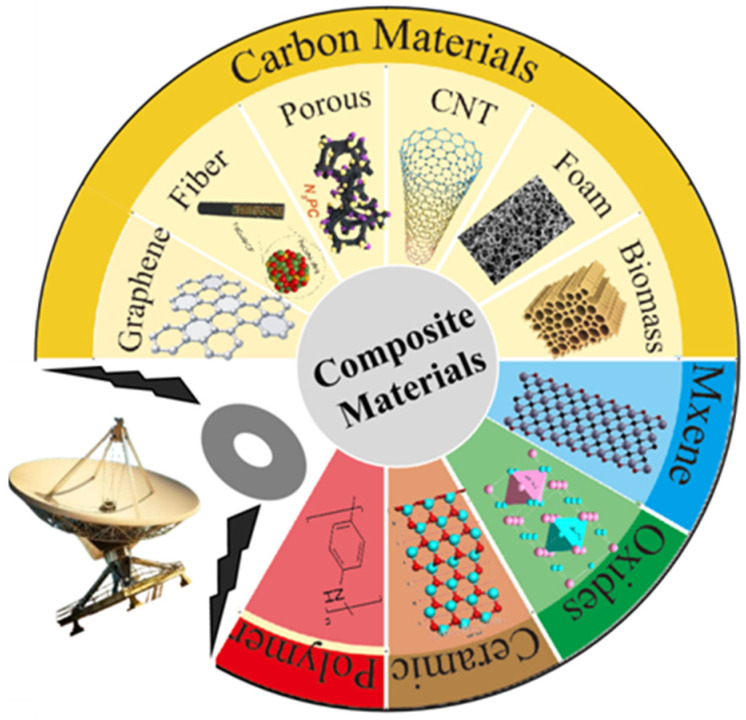
Composition of composite material systems.

As shown in Figure 5 and Table 1, conventional alloy systems such as FeCo and FeNi can be effectively applied in the Ku-band at ultrathin thicknesses of 1–2 mm. In comparison, HEAs like FeCoNiCuC exhibit a significantly enhanced ${RL}_{min}$, reaching values as low as −65.4 dB. Structural designs such as Fe@Ni core-shell nanowires further improve performance by integrating multiple absorption mechanisms, demonstrating excellent microwave absorption across the 2–12 GHz range. For low-frequency applications, large flake-shaped structures demonstrate remarkable performance, such as flaky FeSiAl and FeNi with their respective ${RL}_{min}$ occurring at 1.0 and 0.8 GHz.

## 3. Composite Engineering

Constructing composite materials has emerged as a key strategy for overcoming the persistent challenges of traditional magnetic absorbers. Incorporating functional phases, including carbon materials, MXene, oxides, and conductive polymers, enables the composite to exhibit superior microwave absorption properties. This enhancement originates from the concurrent improvement in both impedance matching and loss capabilities. The categories of composite materials are summarized in Figure 5.

This section will systematically review and discuss the research progress of the aforementioned composite systems, with a focus on their structural design concepts and performance enhancement mechanisms.

### 3.1. Composite Material System

#### 3.1.1. Carbon-Based Composite

Carbon materials [60] offer significant potential for microwave absorption due to their low density, tunable electrical conductivity, and excellent chemical stability. However, their high intrinsic permittivity often leads to impedance mismatch, limiting practical application. By combining carbon materials with magnetic components, both impedance matching and attenuation characteristics can be synergistically optimized.

Carbon nanotubes (CNTs) offer a 1D hollow structure, high aspect ratio, and high conductivity. Through surface modification, magnetic metals/alloys can be introduced into CNTs [61,62,63]. Studies have demonstrated that multi-walled carbon nanotubes (MWCNTs), after electroless plating with an FeCo alloy, exhibit more optimized electromagnetic properties [61]. In addition, CNT/FeSiAl composites [64], constructed by in situ growing of CNTs on FeSiAl flakes, allow optimization of CNT size and quantity through control of the reaction temperature and duration. This composite achieved an EAB of 3.52 GHz and an ${RL}_{min}$ of −47.32 dB at 1.7 mm thickness.

Magnetic carbon fibers, prepared via electrospinning, the hydrothermal–calcination process, electroplating, or magnetron sputtering, exhibit strong and broadband absorption under thin-layer conditions [65]. Examples include CNF-Fe [66], porous P-CNF/Fe [67], FeNi @CNFs [68], FeCoNi@CNFs [69] and so on. FeCo-filled carbon nanofibers (Co/Fe = 1:1) synthesized via a hydrothermal–calcination method [70,71] exhibit an ultra-wide EAB of 12.6 GHz, and ${RL}_{min}$ of −117.8 dB, with excellent wide-angle absorption performance. In comparison, Co_3_Fe_7_-coated carbon fibers [69] prepared by electroplating achieve full-band coverage (2–18 GHz) at a matching thickness of 1.7 mm, along with an ${RL}_{min}$ of −48.2 dB. Moreover, a careful balance is required when incorporating highly conductive CNFs. To avoid excessive electrical conductivity and the formation of a detrimental conductive network, their content is optimally maintained at a low level, around 5 wt%.

Graphene (GN) and reduced graphene oxide (RGO) provide a large specific surface area and a unique 2D layered structure, making them ideal substrates for supporting magnetic nanoparticles. For example, α-Fe nanoparticles (≈10 nm) uniformly loaded on graphene via liquid-phase reduction yielded lightweight G/Fe composites with strong absorption [72]. In situ growth of Ni-Co-P alloy [73] microspheres on GN enabled tunable performance; a Ni:Co ratio of 5:5 gave an ${RL}_{min}$ of −57.8 dB at 1.5 mm, while a 9:1 ratio provided an EAB of 3.6 GHz.

**Figure 6:**
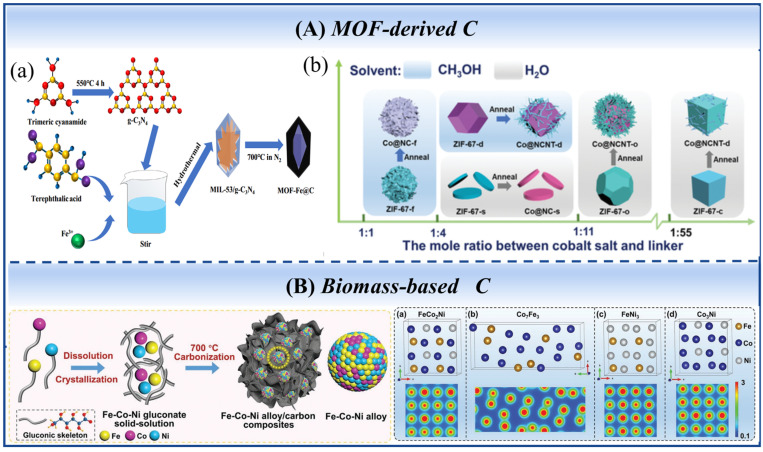
The schematic diagrams of the preparation process and the morphology for (**A**) (**a**) Fe-MOFs, Fe/C porous composite, and (**b**) Co-C porous composite. (**B**) Fe-Co-Ni alloy/biomass-based carbon composite. (Reproduced with permission from Refs. [76,77,83]).

Metal–organic frameworks (MOFs) serve as excellent precursors for magnetic metal/alloy–porous carbon composites, with composition and porosity tunable via precursor design and pyrolysis. ZIF series, MOF-5, and Al-PCPs are widely recognized as ideal templates [74]. For Fe/C composites [75] derived from Fe-MOF, increasing pyrolysis temperature raises porosity, which lowers permittivity and enhances impedance matching. The schematic diagrams of the preparation processes for Fe-MOFs and Fe/C porous composite materials are shown in Figure 6A [76]. According to the Maxwell–Garnett theory, the effective permittivity of the composite is closely related to porosity [75]:

(9) $$\varepsilon_{eff}^{MG}=\varepsilon_{1}\frac{\left(\varepsilon_{2}+2\varepsilon_{1})+2f(\varepsilon_{2}-\varepsilon_{1}\right)}{\left(\varepsilon_{2}+2\varepsilon_{1})-f(\varepsilon_{2}-\varepsilon_{1}\right)}$$

$\varepsilon_{1}$ and $\varepsilon_{2}$ denote the permittivity of the Fe/C composite and air, respectively, while f is the pore volume fraction. The overall permittivity diminishes as the porosity f rises. Furthermore, the generated solid–air interfaces enhance interface polarization and facilitate multiple scattering and reflection of incident microwaves. In addition, by regulating the types of solvents and the molar ratio of magnetic metal to linker, tunable morphologies, from sheet-like, flower-like, cubic, dodecahedral to octahedral, can be obtained [77] (Figure 6A).

Bimetallic MOF-derived composites, such as FeM(II)-alloy@C from FeM-MIL-88B, allow performance tuning through metal ratio adjustment. At Fe:Co = 1:2, optimal microwave absorption performance was achieved at 25 wt% loading [78]. Juan Xiong et al. prepared layered NiCo alloy nanoparticle/nanoporous carbon (NPC) composites [79]. Their investigation identified the carbonization process as the pivotal factor enabling precise control over the composition and microstructure of the NiCo-NPC composites. MOFs@FeSiAl heterostructures [80] constructed via the oxidation-then-compounding strategy achieved an ${RL}_{min}$ of −71.5 dB at 3.85 mm thickness of and realized broadband EAB in the 3–16 GHz range.

Biomass carbon materials (e.g., alginate carbon, straw biochar, and lignin carbon) possess natural porous structures, environmental friendliness, and low cost, making them ideal carriers for magnetic nanoparticles. For example, a viable route to magnetic metal composites involves forming a hydrogel precursor via divalent metal ion crosslinking. Subsequent pyrolysis of this precursor yields the final material. Among these, Ni/Ni_3_ZnC_0.7_/C porous materials [81] achieved an EAB of 5.4 GHz at a thickness of 2.0 mm. Porous FeNi/carbon nanosheets from starch (density 36.3 mg/cm^3^) and FeNi/lignosulfonate-derived composites [82] both exhibit substantial microwave absorption. Fe-Co-Ni/C composites with strong C-band absorption were synthesized through the carbothermal reduction in solid-solution precursors derived from metal gluconates [83]. FeCo_2_Ni/C achieved −82.2 dB at 5.21 GHz, attributed to molecular-level alloy dispersion and magneto-dielectric synergy. First-principles calculations and badger charge analysis revealed that the electronegativity difference between Fe, Co, and Ni atoms causes electron delocalization and dipole polarization. Specifically, electron transfer between Fe and Ni significantly enhances dielectric polarization oscillation and interface polarization. Spin magnetic moment calculations showed that Fe, Co, and Ni all exhibit distinct magnetic moments; their magnetic resonance and antiferromagnetic coupling collectively promote magnetic coupling and magnetic loss. Figure 6B schematically depicts the synthesis process of the Fe-Co-Ni alloy/carbon composites, with the upper and lower sections illustrating the material’s structure and electron density distribution, respectively.

Carbon foams (CFs) provide 3D interconnected porous structures ideal for lightweight and wide-scattering absorption. Among them, nitrogen-doped carbon foam synthesized from melamine foam (CMF) as the precursor can enhance dielectric polarization. With nitrogen doping as the key driver, this further improves the microwave absorption performance. CuNi/CF composites [84] prepared by adsorption–pyrolysis achieved an ${RL}_{min}$ of −50.20 dB at 1.6 mm. Another study employed magnetron sputtering to construct a thin film of (FeNi)_x_(SiO_2_)_1−x_ nanoparticles [85] (≈100 nm thick) on the surface of CF. The FeNi nanograins formed an interconnected network, embedded within an amorphous SiO_2_ matrix. When the SiO_2_ content was 5%, the material achieved an ${RL}_{min}$ of −56.3 dB at 2.5 mm thickness and an EAB of 8 GHz at 2.7 mm thickness. Additionally, further introduction of a SiO_2_ coating layer on the surface of the carbon foam–FeNi significantly improved the low-frequency absorption performance [86].

In summary, carbon materials across different dimensions from 1D to 3D offer a rich design space for developing tailored microwave absorption systems.

#### 3.1.2. Mxene

MXenes (e.g., Ti_3_C_2_T_x_) [87], a class of 2D layered materials, have garnered significant interest in microwave absorption, owing to their high electrical conductivity and rich surface functional groups. However, their high intrinsic permittivity often results in poor impedance matching. A key strategy to address this limitation involves incorporating magnetic components to disrupt the continuous conductive network of MXene.

Flexible MXene-based composite films (5–20 μm thick) have been fabricated by embedding FeCo alloys [88] with varied morphologies (nanoflower-like or nanosheet-like) between MXene interlayers via electrostatic self-assembly and vacuum filtration. The incorporation of FeCo alloys with a nanoflower-like morphology effectively interrupts MXene’s conductive pathways, reducing electrical conductivity from 10^4^ S/m to 10^2^–10^3^ S/m and lowering the $\varepsilon^{\prime }$ from 55 to 15–25. These modifications synergistically improve impedance matching and attenuation capacity, collectively contributing to a marked enhancement in microwave absorption performance. In a similar vein, at a 20 wt% FeNi loading, the FeNi/Ti_3_C_2_T_x_ composites [89] prepared by the in situ hydrothermal method exhibit an EAB of 6.2 GHz at a matched thickness of 1.6 mm. Similarly, the Co/Co_9_S_8_/Ti_3_C_2_T_x_ system, leveraging multidimensional components and heterogeneous interfaces, attains an EAB of 5.36 GHz at 2.1 mm.

#### 3.1.3. Oxide-Based Composite

Oxide-based composite microwave absorbers, constructed by incorporating functional oxides such as Al_2_O_3_ and MgO with magnetic components, enable effective regulation of electromagnetic parameters and demonstrate unique advantages in achieving thermal–wave multifunctional integration.

Among them, net-like ZnO@FeSiAl/silicone rubber composites [90] exhibit a strong ${RL}_{min}$ of −71.38 dB at 2.8 mm, along with an EAB covering the entire X-band. The tensile strength and thermal conductivity of these composites are measured at 3.67 MPa and 1.65 W/(m·K), respectively, demonstrating their balanced mechanical robustness and thermal management potential. ZrO_2_ modification offers similar performance enhancement. Cotton-like ZrO_2_@FeSiAl composites [91], prepared through chemical precipitation and subsequent heat treatment, undergo a morphological transition from chain-like to cotton-like structures as the ZrOCl_2_ content increases. With 4 wt% ZrOCl_2_, the composite achieves an ultra-wide EAB of 9.4 GHz at a thickness of 1.85 mm, spanning the C-, X-, and Ku-bands. MgO@FeSiAl composites also demonstrate excellent microwave absorption across the 8–12 GHz frequency range.

Furthermore, oxide coatings also significantly enhance overall performance. In ZnO-coated FeSiAl systems, flaky FeSiAl/ZnO composites [92] (mass ratio 4:5) exhibit highly efficient microwave absorbing behavior.

#### 3.1.4. Ceramic Matrix Composites

Characterized by superior thermal stability, tailorable dielectric properties, and excellent mechanical integrity, ceramic matrix composites show great promise for utilization under extreme thermal and environmental conditions. Two representative examples are provided below.

Fe/Ni bimetal co-doped SiCN (SiCN/Fe/Ni) ceramics [93] were synthesized via a polymer-derived ceramic route, with interface polarization accounting for over 94% of the total electromagnetic loss. The formation of a multiphase structure comprising C, SiC, Fe_2_Si, Ni_3_Si, γ-(Fe, Ni) solid solution enhances interfacial polarization, while the incorporation of Ni improves electrical conductivity and magnetic loss capability.

Carbon-coated SiC/Fe nanowires [94] prepared by arc discharge exhibit strong GHz-band absorption, reaching an ${RL}_{min}$ of −63.44 dB and an EAB of 7 GHz at 25 wt% filler loading. However, increasing the filler content to 50 wt% leads to impedance mismatch due to excessive permittivity, resulting in degraded absorption performance.

#### 3.1.5. Conductive Polymer Composites

The combination of magnetic materials and conductive polymers (e.g., polyaniline (PANI) and polypyrrole (PPy)) has opened up new research avenues. For instance, in situ polymerization of PPy on Co nanoparticles forms chain-like structures with enhanced interfacial bonding via ligand substitution, leading to strong absorption in the X-band [95]. Kashi et al. [96] found that the $M_{s}$ value decreases with the increase in PANI polymer content in FeNi/PANI nanocomposites. The sample prepared by in situ polymerization exhibits a deficient RL value due to acid erosion; in contrast, the sample prepared by the physical blending method (FeNi:PANI = 1:1) has the widest absorption bandwidth and optimal performance. Beyond this, however, lies a broader research scope centered on the multicomponent hybridization of magnetic materials, conductive polymers, and other dielectric materials.

#### 3.1.6. Multicomponent Composite Microwave Absorbers

Multicomponent composite absorbers represent an advanced direction in microwave absorption research, enabling synergistic integration of multiple loss mechanisms through rational structural and compositional design.

Several advanced composites, including Fe/Fe_3_C@C@PANI [97], biomass-derived Fe@NPC@CF [98], and MOF-templated CoFe@ZnO@C [99], demonstrate that high microwave absorption performance can be achieved under practical conditions of low filler content and thin thickness.

Oxides or ceramics (e.g., SiCN) act as matrices to improve the stability and high-temperature resistance of magnetic materials. For the FeSiAl/flaky graphite/Al_2_O_3_ composite [100], when the FeSiAl particle size is controlled within 25–48 μm, it realizes effective absorption in the X-band even at a thin thickness of 1.0 mm.

Heteroatom doping effectively broadens the absorption bandwidth [101]. Yangjun Zou’s team prepared FeNi/N, S co-doped carbon composites (FeNi/N, S-C) [102] via a microwave-assisted polymerization–sintering process. By optimizing the FeNi content, the composite achieves ultra-broadband absorption performance, with an EAB covering 3.92–17.08 GHz and 18–37.3 GHz (total width = 32.46 GHz). The preparation methods, mass ratios, absorption performance indicators, and matching thicknesses of the representative alloy composite systems are listed in Table 2 below.

### 3.2. Preparation Methods

As summarized in Table 2, a diverse range of synthesis methods has been developed for magnetic microwave-absorbing composites, each demonstrating unique advantages alongside specific constraints. The hydrothermal/solvothermal method [103] enables precise morphology control and good dispersibility (e.g., Fe nanoparticle-decorated rGO), albeit with drawbacks such as long reaction times and challenges in scaling up. Chemical coprecipitation [90] is a simple and scalable route suitable for systems like FeSiAl/oxide composites, though it often leads to particle agglomeration and limited purity. Pyrolysis [104], particularly of MOFs or biomass precursors, allows the one-step formation of porous carbon-based composites with tunable composition, yet involves high energy consumption and potential emission of harmful gases. In contrast to the methods for diverse morphologies discussed earlier, the following techniques focus on thin-film materials. Chemical vapor deposition (CVD) [105] is ideal for growing uniform, adherent films with high precision, such as magnetic particle-embedded CNT arrays, but requires costly vacuum systems and is restricted to thin coatings. Arc plasma technology [106] facilitates the ultra-rapid synthesis of core-shell nanoparticles (e.g., Fe@C) with high purity, though it yields broad size distributions and limited morphological control. Lastly, magnetron sputtering [107] produces highly uniform thin films with accurate stoichiometry, yet remains constrained by low deposition rates and high cost.

**Figure 7:**
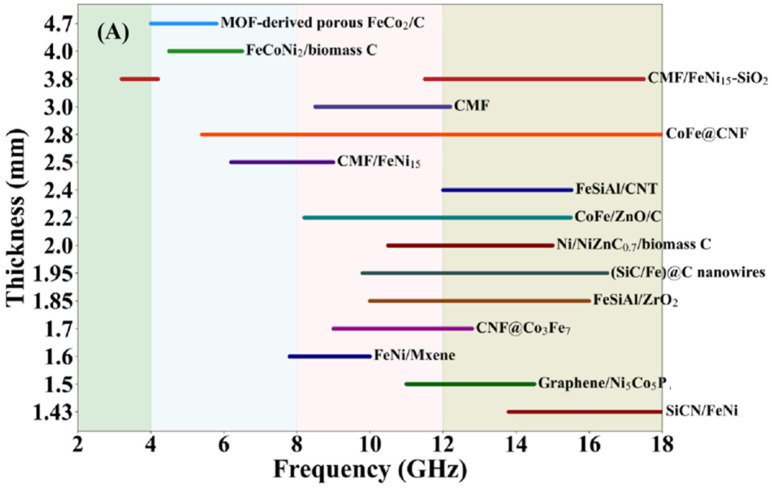

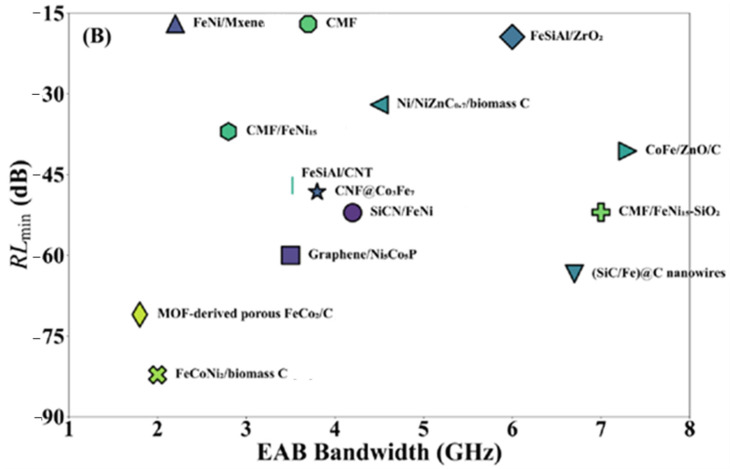
Comparative study of (**A**) EAB and (**B**) ${RL}_{min}$ for representative magnetic composites.

Figure 7 compares the EAB and ${RL}_{min}$ of several representative magnetic composites. We can conclude that simple magnetic–dielectric composites (e.g., with graphene, SiCN, or CNTs) achieve high-performance absorption in the Ku-band at 1.4–2.5 mm thicknesses. Multicomponent systems further push the performance limits. For example, the CoFe@ZnO@C structure significantly broadens the absorption bandwidth. Reference [69] reported a design with metal particles embedded inside carbon fibers, which achieved broadband absorption from 6 to 18 GHz and an ${RL}_{min}$ of −180 dB, substantially outperforming the surface-loaded structure described in Ref. [70]. Notably, in Ref. [86] the CMF substrate itself exhibits intrinsic absorption in the X-band (${RL}_{min}$ ≈ −15 dB). Surface modification with an FeNi layer shifts the absorption to the C-band and deepens the ${RL}_{min}$ to −38 dB. The further addition of a SiO_2_ dielectric layer in the surface extends the bandwidth into the S- and Ku-bands, underscoring the critical role of surface dielectric layers in tuning impedance matching. Meanwhile, porous carbon materials provide stable absorption in the 4–8 GHz range, offering a feasible strategy for designing absorbers tailored to specific frequencies.

### 3.3. Structural Design

In addition, the simple blending of magnetic materials with other substances and structural designs has emerged as a cornerstone of tailoring material properties. This approach spans multiple length scales: from microscale core-shell architectures and precise interface engineering, to hierarchical self-assembly structures, and further extends to macroscale multilayer configurations and the rational design of metamaterials.

#### 3.3.1. Core-Shell Structures

##### Single-Layer Core-Shell Structures

Core-shell architectures, in particular, exhibit remarkable tunability in composition and interfacial properties. This structural design thereby enables synergistic magnetic–dielectric coupling, mitigation of eddy current loss, enhanced interfacial polarization, and improved oxidation resistance [108]. Their high Snoek’s limit further establishes them as a promising platform for high-performance absorber design [109,110].

**Figure 8:**
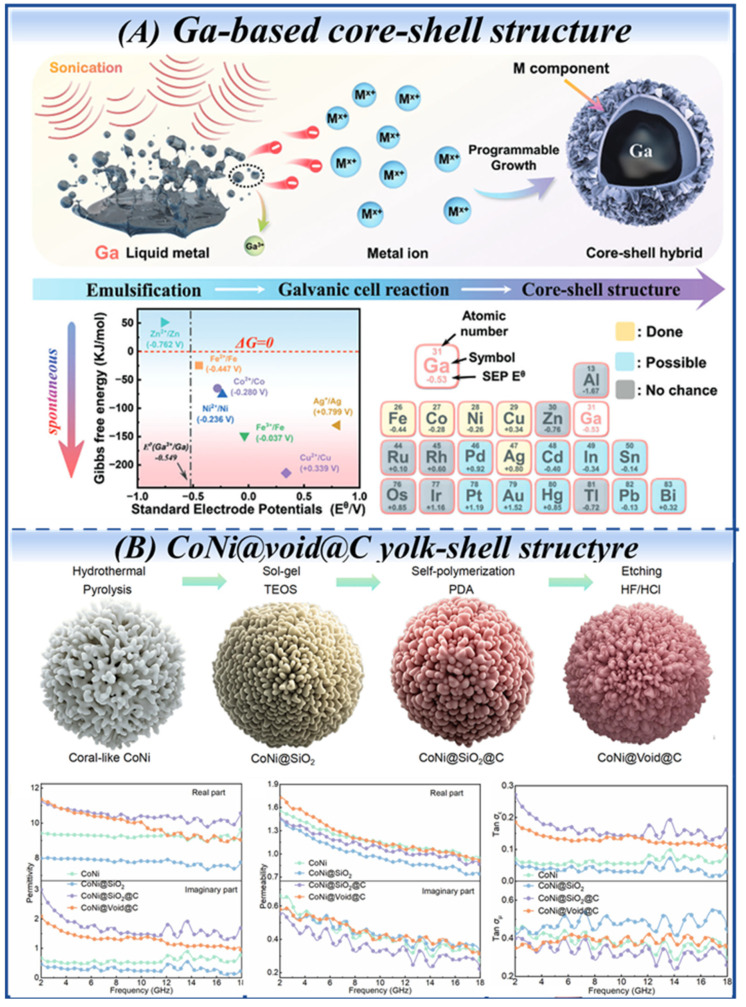
(**A**) Schematic of core–shell hybrid derived from Ga-based liquid metals. (**B**) Structural schematic diagrams and electromagnetic properties of coral-like CoNi, CoNi@SiO_2_, CoNi@SiO_2_@C, and CoNi@void@C. (Reproduced with permission from Refs. [118,124]).

Core-shell nanocapsules typically consist of magnetic cores encapsulated by dielectric shells, such as TiO_2_, ZnO, carbon, or polymers [111]. (Fe, Ni)/C nanocapsules [112] prepared by arc discharge achieve full Ku-band absorption with an optimum value of −26.9 dB at 2.0 mm. Likewise, (Fe_70_Ni_30_)@C, FeNi_3_@C microchains [113], Fe@ZnO [114], Co_7_Fe_3_@C [115], FeCo@SiO_2_ [116] and FeNiMo@C nanocapsules [117] also exhibited both remarkable absorption strength and wide EAB at an ultrathin matching thickness. Notably, Biao Zhao et al. [118] employed liquid-metal Ga alloys as reconfigurable templates to quantitatively regulate the diversity of the heterogeneous nanoparticle shell layer composition, and they demonstrated the initiation of local galvanic replacement reactions utilizing an ultrasonic system. The schematic of the core-shell hybrid derived from Ga-based liquid metals is exhibited in Figure 8A.

In addition, by introducing heteroatoms, the electronic structure, defect concentration, and interface properties of materials can be adjusted. Xinghao Qu et al. synthesized S-doped FeNi@C nanocapsules [119] via in situ doping during arc discharge, leveraging sulfur’s low boiling point. Theoretical analysis confirmed that S substitution created defect-induced dipoles, enhancing both dipole and interfacial polarization, while its role as an n-type dopant improved conductivity via p-electron injection into the graphite π-system. Although S doping slightly reduced $M_{s}$ and increased $H_{c}$, its benefits to dielectric loss dominated, enabling a strong ${\;RL}_{min}$ of −50.3 dB at an ultralow thickness of 1.6 mm. In nitrogen-doped systems [120], the formation of Ni-N bonds in Ni-N@C materials significantly altered the surface electron distribution. By adjusting Ni content, the EAB covered all C-, X-, and Ku-bands (3.90–18.0 GHz), realizing full-band effective absorption.

Core-shell structures not only improve synergistic EMW absorption but also significantly enhance environmental stability (e.g., oxidation resistance and corrosion resistance) [121]. Daubert et al. investigated the protective effects of various oxide films (e.g., Al_2_O_3_, TiO_2_, and HfO_2_) on metallic substrates. Ni/AlN/MWCNT and FeSiAl/h-BN composites [122] offer the additional advantage of excellent corrosion resistance.

##### Yolk-Shell Structures

Yolk-shell structures (core@void@shell configuration) [123] exhibit significant advantages, primarily due to their intermediate cavity. This cavity effectively promotes multiple reflections and scattering of incident EMWs, thereby significantly enhancing energy dissipation and attenuation. In the work of Jiale Wu et al., coral-like CoNi@Void@C microparticles were synthesized via a continuous fabrication process integrating solvothermal, sol–gel, oxidative self-polymerization and acid etching. The dual barrier effect of the cavity buffer layer and nonpolar carbon shell, combined with the material’s electromagnetic balance property, endows it with superior microwave absorption performance compared to other structures like CoNi@SiO_2_ and CoNi@ SiO_2_@C [124] (Figure 8B). A notable innovation was reported in the construction of HEA@air@Ni–NiO yolk-shell microspheres [125] (with an FeCoNiCrCuAl_0.3_ high-entropy alloy core and Ni–NiO shell) using a two-step method involving hydrothermal treatment followed by calcination. An intermediate carbon layer was first formed on the HEA surface, followed by epitaxial growth of a hydroxide precursor and final carbon removal to form the cavity. This structure achieved an EAB of 4.0 GHz at only 1.3 mm.

##### Double Core–Shell Structures

Multishell structures further optimize absorption performance via the coordinated design of diverse components and a graded impedance profile. Co/MnO@C nanocapsules [126] with a magnetic/dielectric dual-core structure were prepared via in situ carbon reduction. After 900 °C annealing, the material achieved an ultra-strong ${RL}_{min}$ of −97.5 dB at 10.6 GHz and an 8.1 GHz EAB (9.9–18 GHz) at 2.1 mm, illustrating an ideal balance between magnetic and dielectric loss. Other double core–shell systems also exhibit superior performance; α-Fe(Si)@Fe_3_O_4_@SiO_2_ [127] enhances absorption (${RL}_{min}$ = −47.04 dB) via interfacial polarization and defect engineering. The Fe@C@BaTiO_3_ sandwich structure [128] reported by Shi et al. exhibited an absorption bandwidth 1.3–2.0 times that of single-shell Fe@C, with an ${RL}_{min}$ of −40.2 dB; this advantage originated from interface polarization and the high dielectric property of BaTiO_3_. FeSiAl@SiO_2_@Polyurethane Acrylate (PUA) inorganic–organic dual-layer composites [129] achieved an ${RL}_{min}$ of −49 dB and a 7.8 GHz EAB at 2.5 mm, while offering excellent corrosion resistance, demonstrating both high absorption and environmental durability.

In addition, multilayer gradient structures and alternating core–shell structures can be constructed at the microscale using magnetron sputtering technology. For example, a five-layer FeNi–SiO_2_ gradient film with a total thickness of 800 nm was deposited on the surface of SiCf [130], in which the SiO_2_ content increases from 0% to 100% with 25% gradient steps. With only 10% mass fraction of SiCf/FeNi–SiO_2_ and a sample thickness of 2.0 mm, the material exhibits a broad EAB of 6.84 GHz, spanning from 11.08 to 17.92 GHz. This performance improvement is attributed to the introduction of the FeNi–SiO_2_ gradient film, which enhances the Debye dipole polarization effect and optimizes impedance matching characteristics.

Through functional layer design, core–shell structures show considerable potential for engineering applications. Future research could focus on the precise regulation of shell structures, in-depth analysis of interface coupling mechanisms, and development of low-cost large-scale preparation processes.

#### 3.3.2. Interface Design Strategies

Beyond the geometric control offered by core–shell structures, microscopic interface engineering represents another vital dimension for performance enhancement. Yihui Zhou et al. reported an Fe/Fe_3_C@C nanocomposite [131] featuring multiple heterogeneous interfaces. The composite was achieved by first growing Fe_3_O_4_ nanoparticles in situ on self-crosslinked polydopamine nanospheres, which were subsequently pyrolyzed at 800 °C.

Wang et al. constructed 1D CNTs on the surface of CoNi nanospheres [132] derived from NiCo layered double hydroxides (NiCo-LDH). DFT calculations revealed that interfacial charge transfer between CNTs and the CoNi matrix enhances electrical conductivity and polarization loss. Density of state (DOS) results further indicated a higher electron density near the Fermi level in the NiCo/CNT composite, suggesting a favorable electronic structure for polarization relaxation. The significant difference in work function between NiCo-LDH (6.26 eV) and CNTs (4.76 eV) drives electron migration from CNTs to NiCo, facilitating the formation of a heterointerface conducive to polarization.

**Figure 9:**
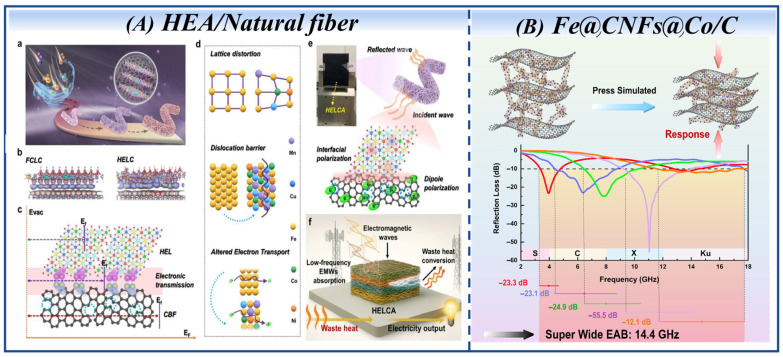
(**A**) Material design concept and expected applications of HELC. (**a**–**c**) Schematic of the synthesis, microstructure, and work function modulation of the HELC heterostructure. (**B**) Schematic diagram of the structure and RL graph of Fe@CNFs@Co/C fibrous elastic aerogel. (Reproduced with permission from Refs. [133,137]).

In the work of Xiaomeng Guan et al. [133], ultrathin high-entropy FeCoNiMnCu layered hydroxide (HEL) nanosheets were uniformly anchored on natural fiber substrates to construct high-entropy interfaces, realizing the synergistic regulation of electromagnetic and thermoelectric loss mechanisms. The high-entropy effect induces electronic redistribution, and elemental heterogeneity enhances the material’s dielectric loss and electromagnetic wave absorption performance via localized polarization and lateral electron migration; lattice-induced strain can also increase the interfacial density and strengthen interfacial coupling effects synchronously. The material design concept and expected applications of HEL integrated with carbon fibers (HELC) are illustrated in Figure 9A.

A strategic bridging approach was implemented, in which the in situ self-polymerization of dopamine served as a bridging platform. This enabled the subsequent growth of a ZIF-derived carbon nanoskeleton on carbonyl iron, ultimately forming multi-heterogeneous Fe/FeCo@C interfaces after thermal treatment [134]. This absorber was then integrated with polypropylene (PP) via melt-blending, enabling its fabrication through FDM 3D printing into an FCC/PP composite. The composite exhibits outstanding performance at a low filler loading of 40%. Similarly, the reported porous Fe/FeO/Fe_2_O_3_ nanorod/RGO composite [135] effectively enhances the synergy between interfacial polarization and magnetic loss.

Through a simple vertical ball milling method, layered FeSiCr particles coated with an Fe_3_O_4_ layer (with embedded ~10 nm FeSiAl nanoparticles) were successfully prepared. Adding Cr to Fe-Si alloys can improve the temperature stability and enhance their ductility, but it also increases the $H_{c}$, leading to a reduction in high-frequency absorption intensity. The construction of abundant FeSiCr/FeSiAl heterogeneous interfaces, achieved by embedding FeSiAl nanoparticles into FeSiCr/Fe_3_O_4_ flakes [136], synergistically combines the complementary advantages of both components. This configuration enables the concurrent realization of strong RL, a wide EAB and a minimal matching thickness, alongside demonstrated thickness stability and a broad radar cross-section (RCS) reduction capability.

As evidenced by the above examples, interface engineering optimizes impedance matching while introducing strong polarization relaxation, multiple scattering, and geometric extension effects, thereby enabling synergy among components.

More advancedly, the Fe@CNFs@Co/C fibrous elastic aerogel [137] prepared via the electrospinning (Fe-MOF@PAN)–impregnation growth (Co-MOF)–heat treatment strategy has opened up a new paradigm for intelligent microwave-absorbing materials. This material exhibits significant pressure response characteristics; under pressure, the fiber layer spacing decreases, the 3D conductive network becomes more complete, and impedance matching is dynamically optimized. Finally, an ultra-wide EAB of 14.4 GHz (3.36–17.76 GHz) is achieved, covering 90% of the S/C/X/Ku-bands, providing a new direction for the design of next-generation intelligent dynamic microwave-absorbing materials. A schematic diagram of the structure and an RL graph of the Fe@CNFs@Co/C fibrous elastic aerogel are illustrated in Figure 9B.

#### 3.3.3. Self-Assembled Hierarchical Structures

**Figure 10:**
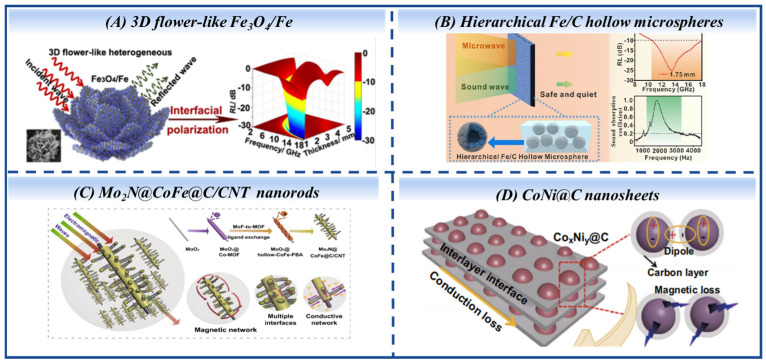
Self-assembled hierarchical structures. (**A**) 3D flower-like Fe_3_O_4_/Fe, (**B**) hierarchical Fe/C hollow microspheres, (**C**) Mo_2_N@CoFe@C/CNT nanorods, (**D**) CoNi@C nanosheets. (Reproduced with permission from Refs. [138,142,145,146]).

The assembly of microscopic lamellar structures is a widely adopted strategy for constructing high-performance MAMs. Xueai Li et al. [138] (Figure 10A) fabricated 3D flower-like heterogeneous Fe_3_O_4_/Fe particles with Fe_2_O_3_ as a sacrificing template; the chemical composition of the particles can be flexibly regulated via the reduction temperature, thereby realizing the modulation of their electromagnetic and reflection loss characteristics. In addition, hierarchical rose-like porous Fe@C composites [139], porous flower-like Co@CoO nanohybrids [140] and Nix/C heterostructures [141] demonstrate remarkable microwave absorption.

Bao Susu et al. reported the synthesis of hierarchical Fe/C hollow microspheres [142] (Figure 10B). The composite features a spherical shell self-assembled from centripetally aligned lamellae, composed of a carbon matrix embedded with variably oriented iron particles. This configuration results in an outward-loosely and inward-tightly packed architecture. It exhibited efficient microwave and sound absorption, highlighting its potential as an integrated microwave–acoustic absorption material. Porous microspheres self-assembled from core–shell nanorods constitute the Ni/NiO architecture [143], which is synthesized through a combined hydrothermal and thermal reduction approach. The substantial specific surface area and the profusion of NiO–Ni interfaces are instrumental in optimizing the impedance matching characteristics, yielding an ${RL}_{min}$ of −52.15 dB and a 3.22 GHz bandwidth at 2.17 mm.

Lei Wang et al. [144] successfully prepared hierarchical nest-like Co/Fe@C composites assembled from porous CoFe@C nanorods by annealing CoFe-MOF-74 at 800 °C in an argon atmosphere with a Co/Fe molar ratio of 3:1. The composite demonstrated exceptional microwave absorption performance at a low filler loading of 10 wt%. Similarly, Renchao Che’s research group [145] successfully fabricated a three-dimensionally hierarchical microrod-supported nanotube-type core–shell magnetic metal–carbon composite. Magnetic CoFe nanoparticles were embedded in one-dimensional graphitized C/CNTs supported on microscale Mo_2_N rods, yielding the multidimensionally hierarchical microwave-absorbing material Mo_2_N@CoFe@C/CNT (Figure 10C). This material achieves a maximum reflection loss of −53.5 dB and an effective absorption bandwidth of 5.0 GHz at a thickness of 2 mm.

In another study, stacked Co_x_Ni_γ_@C nanosheets (Figure 10D) were fabricated via carbonization of a stacked CoNi-MOF template [146]. The continuous carbon layers facilitate electron transport, while the multi-interface and porous structure enhance polarization loss. These features collectively strengthen the dielectric loss capability. Specifically, with a Co^2+^ to Ni^2+^ feeding ratio of 1:1, the resulting CoNi@C nanosheets exhibited an EAB of 5.7 GHz at 1.8 mm, covering key frequencies relevant to electromagnetic protection. Fluffy Co/CoO micro-rod composites [147] were produced through a solution-based route and subsequent annealing, where the final cobalt content was precisely regulated by the annealing temperature. The sample annealed at 500 °C and further treated at 700 °C under nitrogen showed outstanding microwave absorption, achieving an ${RL}_{min}$ of −21.7 dB and an EAB exceeding 6.1 GHz at a thickness of 2.3 mm. The preparation methods and key microwave absorption performance metrics of the structurally designed composite materials are summarized in Table 3.

Through a relatively simple and controllable process, the self-assembly approach achieves its core advantage by integrating nanounits (e.g., nanorods and nanosheets) into complex 3D micro-architectures (e.g., nest-like, flower-like, hollow spheres). This process spontaneously creates hierarchical, multi-interface microstructures, thereby enabling precise multiscaled structural control.

#### 3.3.4. Macrostructural Design (Multilayer Structure and Metamaterial)

Multilayer microwave-absorbing materials demonstrate exceptional design flexibility, enabling the creation of tailored impedance gradients that maximize microwave attenuation, broaden the absorption bandwidth, and overcome the narrow frequency response limitations inherent in conventional single-layer absorbers. Consequently, they have become a major research focus in the field.

Xin Sun et al. [148] successfully fabricated a high-performance microwave absorber with an ultra-wide EAB of 12.85 GHz. Its exceptional performance is attributed to an elaborate multilayer structural design, which integrates a wave-transparent layer, a carbon–magnetic composite absorption layer (filled with hollow Co/CoFe@C composites), a strong magnetic absorption layer (composed of FeSiAl and SmFeB), and a reflective layer. This multicomponent, multi-mechanism synergistic design strategy effectively overcomes the bandwidth limitation of conventional single-layer absorbers, thereby fully demonstrating its structural advantages.

Li He et al. [149] employed the Particle Swarm Optimization (PSO) algorithm to design multilayer magnetic–carbon composites. Initially, four single-layer absorbers were fabricated by incorporating Fe_3_S_4_ hollow microspheres and carbon nanotubes as functional fillers. Through systematic optimization, a double-layer structure with a thickness of 3.8 mm was identified as the optimal configuration, exhibiting a simulated ${RL}_{min}$ of −40 dB and an EAB of 11.7 GHz.

**Figure 11:**
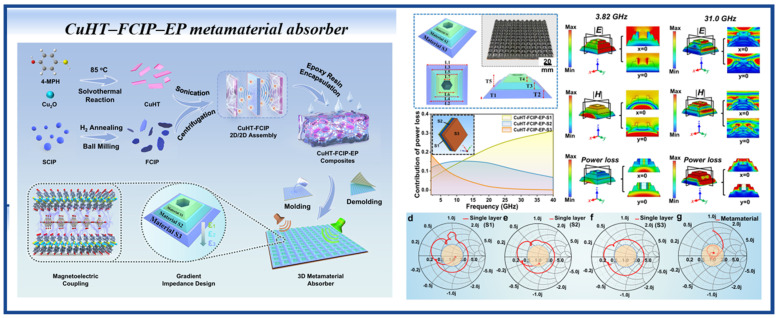
Schematic illustration of the preparation of CuHT–FCIP–EP metamaterial absorber. (Reproduced with permission from Ref. [151]).

Metamaterial design represents an emerging structural optimization strategy for microwave absorbers, achieving breakthroughs in broadband and high-efficiency absorption through the integration of intrinsic material properties and artificial structural resonance effects. This approach provides novel pathways to overcome the limitations of traditional absorbing materials. In the CNT/FeNi composite system [150], a single-layer structure achieved an EAB of 6.2 GHz at a thickness of 2.5 mm, while a three-layer gradient structure further expanded the bandwidth to 14.7 GHz, covering 3.3–18 GHz. Ning Qu et al. [151] fabricated conductive MOF (SC-MOF)-based 2D microsheets (CuHT), which were hybridized with flake-layered carbonyl iron powders (FCIPs) to construct a sandwich-like 2D/2D assembly (CuHT–FCIP). Subsequently, this assembly was integrated into a novel 3D metamaterial structure that combines the dual advantages of gradient impedance design and a honeycomb perforated structure (Figure 11). This composite material not only achieves an ultra-broad effective absorption bandwidth (EAB) of 2–40 GHz, but also exhibits ultra-wide oblique incidence adaptability within 75° and high polarization insensitivity. Surface energy flow densities and electric and magnetic field intensities reveal that the formation of absorption peaks at different frequency bands is mainly attributed to the thickness resonances at λ/2 and λ, as well as the resonance effects of the bottom layer and honeycomb structure. The impedance matching plot further confirms that the CuHT–FCIP–EP-based metamaterial achieves near-ideal impedance matching, a characteristic unattainable for single-component metamaterials.

In the research from Debao Fang’s group [152], based on MOF-derived prismatic core–shell In/C@Co/C-x composites, the design of a series of periodic stepped microwave-absorbing structures was performed with the aid of HFSS software. The four-layer stepped structure achieved an ultra-wide EAB of 15.28 GHz, covering 95.5% (0.955) of the target frequency range. Mengchao Guo et al. [153] improved the brittleness of FeCo alloys via Ge doping, and designed a metamaterial while maintaining the Curie temperature of the alloy at 850.8 °C. This metamaterial achieves ultra-broadband microwave absorption in the range of 6.8–60 GHz with a total thickness of only 2 mm. Chuyang Liu et al. [154] fabricated an elastic microwave absorber possessing a dome-shaped array structure by integrating NiCo chains and functionalized carbon nanotubes (CNTs-OH) into a platinum-catalyzed silicone rubber matrix. Under progressively increasing compressive strain, the macroscopic dome arrays gradually flatten, thereby altering the propagation path of incident microwaves. Concurrently, the reduced interlayer spacing between isolated micro-conductive networks promotes the formation of additional conductive pathways, leading to the modulation of equivalent electromagnetic parameters. A continuous shift in the reflection loss peak from 11.8 GHz to 9.4 GHz is observed as the applied compressive strain increases from 0% to 25%, demonstrating dynamically tunable microwave absorption behavior. These findings demonstrate considerable application potential in advanced radar stealth technologies, particularly for high-speed aircraft subjected to dynamic aerodynamic pressure.

Artificial intelligence algorithms have been applied to the design of metamaterials to accelerate the development process. In a study by M. Feng et al., the deep integration of structural bionics, intelligent algorithms, and multiphysics coupling has enabled the efficient synergistic optimization of microwave absorption bandwidth, mechanical strength, and structural thickness [155]. The resulting electromagnetic absorbing structure exhibits outstanding integrated performance: a thickness of only 9.3 mm, an EAB of 36.4 GHz covering 2–40 GHz, an equivalent flexural strength of 334.3 MPa, and a compressive strength of 83 MPa. Inspired by the natural antireflection photonic structure of the Polygonia c-aureum butterfly’s eye, specifically, an artificial neural network (ANN) was employed to construct surrogate models. These models were integrated into a heuristic multi-population genetic algorithm (MPGA). Multilayer structure and metamaterial design [156] represent two pivotal strategies for overcoming the performance limitations of traditional absorbing materials and achieving the thin, lightweight, wideband, and strong absorption goal. Specifically, the fundamental role of multilayer design lies in constructing precise impedance gradients and loss distributions, whereas the innovative capacity of metamaterial design resides in introducing structural resonance effects that surpass intrinsic material properties.

A brief summary is presented below on the structural design of magnetic composite materials. At the microscale, core–shell structures formed by coating dielectric layers onto magnetic particles improve both surface impedance matching and interfacial polarization loss. Yolk–shell architectures further extend the electromagnetic wave propagation path via their distinctive cavity effect, which promotes multiple reflections and scattering. Multi-core–shell systems establish continuous impedance variations through carefully engineered spatial composition gradients, enabling adaptive and broadband electromagnetic dissipation.

At the fabrication level, interface engineering allows atomic-/nanoscale control over heterointerface chemistry and defect states, markedly intensifying polarization relaxation and broadening the frequency response. Self-assembled structures, driven by intermolecular interactions, spontaneously organize nanoscale building blocks into ordered mesostructures, permitting synergistic tuning of porosity, specific surface area, and interface density. This offers an ideal platform for coupling diverse loss mechanisms.

On the macroscale, multilayer structures broaden the absorption bandwidth by strategically stacking functional layers with tailored electromagnetic parameters and thicknesses, achieving frequency complementarity and phase synergy. Metamaterials transcend the intrinsic limitations of natural substances by employing resonant subwavelength units that produce strongly localized magnetic fields and electric resonances at targeted frequencies, thereby creating pathways to low-frequency, ultrathin absorption.

In summary, structural design has emerged as a comprehensive and hierarchical strategy that bridges atomic-scale ordering and macroscopic architecture. By integrating multiscale modeling techniques, such as density functional theory for interface properties, phase-field simulations for mesostructural evolution, and machine learning-enabled inverse design of macroscopic layouts, a systematic framework is being established that connects microscopic mechanism analysis with macroscopic performance customization. This integrated approach lays a solid foundation for the development of a new generation of high-performance, multifunctional absorbing materials.

## 4. Conclusions and Outlook

### 4.1. Conclusions

(1) From the perspective of loss mechanisms, this study focuses on synergistic regulation, identifying alloy design, composite engineering, and structural engineering as the core strategies to overcome the inherent limitations of magnetic materials. Regarding alloys, this work systematically expands the alloy design paradigm from binary alloys and high-entropy alloys to tailored elemental doping systems, and clarifies the quantitative regulatory effects of grain size, phase composition, and crystallinity on absorption properties.

(2) This study emphasizes that the synergistic construction of material systems with function-oriented customization is a critical foundation for improving microwave absorption performance. Carbon materials offer advantages such as low density and tunable electrical conductivity, the combination with magnetic materials effectively balances impedance matching and electromagnetic attenuation efficiency. Furthermore, MXene/conductive polymers are suitable for flexible absorbing films, while ceramic-based materials offer excellent stability and corrosion resistance in high-temperature harsh environments.

(3) Multiscale structural design is confirmed as a core approach to achieve broadband and strong absorption. At the microscale, core–shell structures and interface engineering can suppress eddy currents and enhance absorption performance through multi-interface polarization and cavity scattering effects. Hierarchical self-assembled structures integrate porosity, core–shell configurations, and morphological effects, enabling extremely low reflection loss at ultrathin thicknesses. At the macroscale, multilayer structures and metamaterial designs leverage resonance and diffraction effects to break through the bandwidth limitations of conventional materials. Moreover, the structural design paradigm has been profoundly advanced by computational tools, ranging from DFT and finite element simulations to data-driven artificial intelligence.

(4) This study further reveals the significant role of the preparation and post-treatment synergistic regulation in determining the final absorption performance of materials, with a unique focus on the targeted optimization of post-treatment processes. Annealing and magnetic field treatments primarily regulate crystal phase and crystallinity, while surface modifications significantly enhance environmental stability without compromising absorption performance.

### 4.2. Outlook

As pivotal functional components, magnetic materials still face significant challenges that hinder their widespread application. First, the tunability of P-band magnetic properties under ambient conditions is limited; thermal instability in high-temperature environments restricts their L-band performance near the Curie temperature. These issues necessitate further efforts through optimized composition design and heterostructure engineering. Furthermore, density constraint issues remain core bottlenecks in lightweight application scenarios, such as aerospace and portable electronics, and long-term stability is also a matter of concern. From a practical standpoint, the cost burden associated with complex synthesis routes severely impedes large-scale industrial deployment, highlighting the urgent need to develop low-cost alternatives or resource-efficient fabrication strategies. Additionally, the scalable synthesis of high-performance magnetic materials still demands the development of reproducible, eco-friendly processes to ensure batch consistency and minimize environmental impact.

## Figures and Tables

**Table 1:** The preparation methods, mass ratios, microwave absorption parameters, and matching thicknesses of representative alloy systems.

Sample	Preparation Method	Mass Ratio	Absorber Thickness (RL < −10 dB) (mm)	Frequency Range (RL < −10 dB) (GHz)	Minimum RL			Ref
					Value (dB)	Matching Thickness (mm)	Frequency (GHz)	
Fe_20_Ni_80_	Liquid-phase reduction	80%	1.0	12.0–16.0	−17.0	1.5	14.5	[27]
Fe_7_Co_3_	Liquid-phase reduction	70%	1.6	10.0–18.0	−56.0	1.6	14.2	[28]
Ni_2_MnGa	Arc melting	66.7%	1.8–2.8	9.0–18.0	−46.0	2.8	13.2	[30]
FeCoNiCrAl	Mechanical alloying	70%	1.0–3.5	4.0–18.0	−36.0	1.5	10.2	[31]
FeCoNiCr_0.4_Mn_0.6_	Ball milling	60%	1.0–4.0	2.0–18.0	−48.0	2.8	6.42	[32]
FeCoNiCuC_0.37_	Ball milling	70%	1.5–5.0	3.0–18.0	−65.4	2.2	12.0	[33]
FeSiAl-_3_Gd	Vacuum sintering	50%	2.0–5.0	3.0–18.0	−54.6	3.5	6.0	[35]
Flaky Fe_60_Ni_40_	Mechanical alloying and annealing	40%	2.0–5.0	0.7–2.5	−23.0	5.0	0.8	[41]
Spherical FeSiAl		50%	2.7–5.0	4.5–16.0	−22.0	2.7	14.0	[42]
Flaky FeSiAl	Ball milling	50%	3.0–6.5	1.0–2.5	−18.0	6.5	1.0	[42]
Dendritic FeNi	Electroplating	60%	1.5–5.0	3.5–18.0	−39.0	2.3	9.5	[46]
Fe_15_(Co_0.2_Ni_0.8_)_85_ ternary Janus particles	Confined liquid–solid redox reaction	75%	1.5–3.5	4.0–18.0	−38.0	2.5	8.2	[47]
Fe@Co nanoparticles	Electroless plating	70%	1.3–3.0	6.5–18.0	−22.0	1.4	17.5	[49]
Fe@air@Co nanoparticles	Electroless plating	70%	1.1–3.0	5.5–18.0	−42.8	1.6	11.0	[49]
Fe@Ni_40_ nanowires	Liquid-phase reduction	25%	2.0–5.0	3.0–18.0	−41.6	2.9	7.0	[16]
Ag@Ninanowires	In situ growth	70%	2.0–5.0	4.0–18.0	−61.1	2.9	12.8	[48]
Fe-_25_Cr-_12_Co	Stepwise aging and chilling	90%	8.0–23.5	0.3–1.8	−35.2	15.7	0.7	[53]
Pr–Fe–Ni	Arc melting and high-energy ball milling	80%	1.5–3.5	2.2–8.5	−23.0	3.5	2.8	[54]
CoNi nanosphere	Liquid-phase reduction	40%	3.5–5.0	10.0–18.0	−22.0	4.0	12.0	[58]
CoNi nanosphere	Liquid-phase reduction method and magnetic field	40%	3.5–5.0	7.0–15.2	−38.0	3.0	14.0	[58]
Fe_50_Ni_50_	Liquid-phase reduction and transverse magnetic field treatment	80%	1.5–5.0	2.0–18.0	−30.0	2.0	2.0	[59]

**Table 2:** The preparation methods, mass ratios, absorption performance indicators, and matching thicknesses of representative alloy composite systems.

Sample	Preparation Method	Mass Ratio	Absorber Thickness (RL < −10 dB) (mm)	Frequency Range (RL < −10 dB) (GHz)	Minimum RL			Ref
					Value (dB)	Matching Thickness (mm)	Frequency (GHz)	
FeSiAl-SWCNT	Catalytic chemical vapor deposition	40%	1.5–5.0	3–18	−52.0	2.5	6	[64]
CNFs/Fe	Electrospinning	5%	1.1–5.0	3–18	−67.5	1.3	16.2	[66]
CNFs/Co	Electrospinning	5%	1.1–5.0	3.2–18	−63.1	1.6	15.2	[66]
CNFs/Ni	Electrospinning	5%	1.1–5.0	3.5–18	−61.0	1.7	15.5	[66]
Porous-CNF/Fe	Electrospinning	20%	1.5–4.5	3.8–15.8	−44.9	4.1	4.8	[67]
CoFe@CNF	Hydrothermal self-polymerization	50%	2.8	6–18	−180.0	2.8	11.8	[70]
Fe/graphene	Chemical synthesis	20%	2.0–8.0	3–18	−47.0	7	4.5	[72]
Ni_5_Co_5_P/RGO	Hydrothermal method	20%	1.5–4.5	3.8–13.8	−57.8	1.5	12.2	[73]
Fe/C MOF-Derived Porous Carbon	Solvothermal reaction combined with in situ pyrolysis process	30%	1.5–5.0	3.5–17.8	−37.6	1.5	4.0	[75]
FeCo_2_/MOF-Derived Porous Carbon	Pyrolysis	25%	1.0–5.0	3.8–18.0	71.4	4.7	4.2	[78]
NiCo/MOF-Derived Porous Carbon	In situ pyrolysis	30%	1.5–1.8	12.0–18.0	−34.0	1.5	14.8	[79]
FeSiAl/MOF-Derived Porous Carbon	Oxidative heat treatment	80%	1.5–5.0	3.0–18.0	−37.0	3.0	7.8	[80]
Ni/Ni_3_ZnC_0.7_/Calginate-Derived Carbon	Freeze-drying and carbonization	16.7%	2.0–5.5	4.0–18.0	−42.0	2.0	14.8	[81]
Fe-Co_2_-Ni alloy/Biomass-Based Carbon	Carbothermal reduction	50%	1.5–5.0	4.2–18.0	−82.2	4	5.2	[83]
FeNi/lignosulfonate carbon	Hydrothermal and carbonization	50%	1.0–5.2	5.8–18.0	−55.3	1.5	11.9	[82]
CuNi/CF	Chemical synthesis	40%	1.7–4.0	3.5–13.5	−50.0	2.0	11.0	[84]
CMF/(FeNi)_95_(SiO_2_)_5_	Magnetron sputtering	50%	2.0–5.0	4.5–18.0	−56.3	2.5	13.8	[85]
CMF/FeNi_15_-SiO_2_	Magnetron sputtering	50%	1.5–5.0	2.5–12.0	−53.0	3.0	3.4	[86]
Mxene/FeCo	Electrostatic self-assembly and vacuum-assisted filtration	50%	1.5–5.0	2.2–8.0	−46.0	3.5	6.0	[88]
FeNi/Ti_3_C_2_Tx MXene-2	In situ hydrothermal method	20%	1.2–2.0	8.2–18.0	−25.0	1.4	18.0	[89]
FeSiAl/ZnO	Coprecipitation	50%	1.0–5.0	2.0–18.0	−33.0	1.0	14.7	[90]
(SiC/Fe)@C nanowires	Plasma arc discharge	25%	1.5–5.0	3.6–18.0	−63.4	1.9	12.1	[94]
SiCN/Fe/Ni	Polymer-derived ceramic	70%	1.5–5.0	3.0–18.0	−22.0	4.0	5.0	[93]
CoFe@ZnO@C	In situ growth	30%	2.2–5.0	4.0–18.0	−46.0	5.0	5.6	[99]
CNT/FeSiAl Hybrid Flake/Al_2_O_3_	Catalytic chemical vapor deposition	40%	1.2–5.0	3.0–18.0	−48.0	2.5	9.1	[100]
FeNix/NS-C	Microwave-assisted method	16.7%	1.0–5.5	3.9~18.0	32.0	3.5	8.5	[102]

**Table 3:** The preparation methods, mass ratios, microwave absorption parameters, and matching thicknesses of representative structurally designed composite material systems.

Sample	Preparation Method	Mass Ratio	Absorber Thickness (RL < −10 dB) (mm)	Frequency Range (RL < −10 dB) (GHz)	Minimum RL			Ref
					Value (dB)	Matching Thickness (mm)	Frequency (GHz)	
(Fe, Ni)/C nanocapsule	Vapor Arc Discharge	40%	1.7–2.1	11.5–18.0	−26.9	2.0	14.0	[112]
Fe/ZnO	Low-Temperature Wet Chemical	50%	1.6–2.5	8.0–16.0	−48.6	1.6	15.8	[114]
Co_7_Fe_3_@C	Crystallization–Carbonization–Reduction	50%	1.3–2.3	6.0–18.0	−117.4	1.6	12.0	[116]
FeNiMo@C	Arc Discharge	40%	1.4–2.5	5.0–18.0	−64.0	1.9	13.2	[117]
FeNi@C(S2.5) NCs	DC (Direct Current) Arc Discharge Plasma	50%	1.0–3.1	6.0–18.0	−50.0	1.6	14.2	[119]
Flaky FeSiAl@h-BN	Plasma Ball Milling	50%	1.2–5.0	3.0–18.0	−55.7	2.1	14.0	[122]
HEA@AIR@nI-NIO	Hydrothermal Reduction	50%	1.3–5.0	3.0–18.0	−45.0	1.3	16.2	[125]
Co/MnO@C	Nitrate Pyrolysis and Carbon Reduction	50%	1.4–4.0	5.0–18.0	−100.0	2.6	10.2	[126]
α-Fe(Si) @Fe3O4@SiO2	High-Temperature Mechanochemical Method	70%	1.2–5.0	2.5–18.0	−50.2	5.0	2.9	[127]
Fe@C@BaTiO3	Arc Discharge Plasma Method Combined with Sol Method	60%	1.4–7.0	2.0–18.0	−42.0	2.2	8.0	[128]
FeSiAl@SiO2@PUA	In Situ Polymerization Sol–Gel Method	20%	2.5–5.0	5.0–18.0	−49.0	2.3	6.0	[129]
NiCo/CNT composites	Catalytic Carbonization	20%	1.5–4.0	5.0–18.0	−42.0	1.5	14.0	[132]
FeCo-phenolic-based carbon aerogels	Sol–Gel, Freeze-Drying and High-Temperature Annealing	20%	1.5–5.0	4.0–18.0	−58.5	2.1	13.0	[134]
Fe3O4-coated FeSiCr	Ball Milling	60%	1.0–6.0	3.0–18.0	−30.0	4.5	3.0	[136]
Hierarchical Fe@CNFs@Co/C elastic aerogels	Electrospinning and Heat Treatment.	15%	1.6–4.8	3.0~18.0	−55.5	2.4	11.0	[137]
Hierarchical rose-like Fe@C	In Situ Synthesis	50%	1.0–5.0	2.2–18	−71.5	1.5	8.0	[139]
CoFe@C hierarchical nest-like structure	Pyrolysis	10%	2.0–4.0	7.0–18.0	−63.0	2.8	12.8	[144]
CoNi@C nanosheets	Carbonization	20%	1.2–5.0	4.0–18.0	−42.0	1.7	15.0	[146]
Fluffy Co/CoO micro-rod composites	Hydrothermal and Pyrolysis	60%	1.8–4.0	6.0–10.0	−10.8	4.0	3.9	[147]

## Data Availability

No new data were created or analyzed in this study. Data sharing is not applicable to this article.

## References

[1] Ren J., Mu Z., Sellami R., El-Bahy S.M., Liang G., Guo J., El-Bahy Z.M., Xie P., Guo Z., Hou H. (2025). Multifunctions of microwave-absorbing materials and their potential cross-disciplinary applications: A mini-review. Adv. Compos. Hybrid Mater..

[2] Liu C., Feng Y., Sun H., Hu Q., Xiao H., Guo X., Zhang H. (2025). High-performance SiBCN/Fe-Co-Ni fibers: From broadband absorption to stealth antenna design. Compos. Part B Eng..

[3] Qin M., Zhang L., Wu H. (2022). Dielectric Loss Mechanism in Electromagnetic Wave Absorbing Materials. Adv. Sci..

[4] Abbas S., Chandra M., Verma A., Chatterjee R., Goel T. (2006). Complex permittivity and microwave absorption properties of a composite dielectric absorber. Compos. Part A Appl. Sci. Manuf..

[5] Zhou Y., Chen L., Jian M., Liu Y. (2022). Recent Research Progress of Ferrite Multielement Microwave Absorbing Composites. Adv. Eng. Mater..

[6] Meng X., Xu W., Ren X., Zhu M. (2024). Progress and Challenges of Ferrite Matrix Microwave Absorption Materials. Materials.

[7] Yang Y., Hassan S.U., Zai M., Shah M., Zafar S., Hou L., Wang S. (2024). Compositional design of C-coated multi-elemental alloy nanoparticles for superior microwave absorption. J. Alloys Compd..

[8] Zhang Y., Zhang Y., Yan L., Liu R., Liu C., Wu F., Liu X., Miao X., Shao Y., Gong Y. (2024). Nd-, La-induced precipitate/defect in cobalt-iron magnetic alloy for strong and broadband microwave absorption. Acta Mater..

[9] Zha B., Tao J., Zhou J., Tao X., Zou K., Yao Z. (2024). Enhancing microwave absorption via ion exchange-regulated metal elemental-alloy transition behavior. Chem. Eng. J..

[10] Nan K., Zhao Y., Wang B., Yin S., Peng Y., Huang J., Zhang S., Lei T., Wang Y., Yang Z. (2025). Synergistic multiscale architecture design and heterointerface engineering enable tailored electromagnetic wave absorption and multifunctional integration. Carbon.

[11] Barba A., Clausell C., Jarque J., Nuño L. (2020). Magnetic complex permeability (imaginary part) dependence on the microstructure of a Cu-doped Ni–Zn-polycrystalline sintered ferrite. Ceram. Int..

[12] Pang H., Fan M., He Z. (2012). A method for analyzing the microwave absorption properties of magnetic materials. J. Magn. Magn. Mater..

[13] Zuo D., Jia Y., Xu J., Fu J. (2023). High-Performance Microwave Absorption Materials: Theory, Fabrication, and Functionalization. Ind. Eng. Chem. Res..

[14] Elmahaishi M.F., Azis R.S., Ismail I., Muhammad F.D. (2022). A review on electromagnetic microwave absorption properties: Their materials and performance. J. Mater. Res. Technol..

[15] Zarkevich N.A., Nlebedim C.I., McCallum R.W. (2021). Parameterization of the Stoner-Wohlfarth model of magnetic hysteresis. J. Magn. Magn. Mater..

[16] Cai R., Yang P.-A., Ruan H., Huang X., Zhou Z., Chen Q., Zhang Y., Gong X., Gui Y., Li R. (2024). Directional control of electromagnetic parameters of Fe@Ni nanowires for ultrathin and low-frequency microwave absorbing. Appl. Surf. Sci..

[17] Wang S., Zhang W., Zhang Y., Zhao J., Li R., Zhong Y. (2024). Effect of Reduced Graphene Oxide on Microwave Absorbing Properties of Al_1.5_Co_4_Fe_2_Cr High-Entropy Alloys. Entropy.

[18] Zhang S., Lan D., Zheng J., Zhao Z., Jia Z., Wu G. (2024). Insights into polarization relaxation of electromagnetic wave absorption. Cell Rep. Phys. Sci..

[19] Li Y., Yang J., Huang S., Deng L., He L. (2024). Design and Broadband Absorption Properties of a Composite Metamaterial Microwave Absorber Based on the Debye Dielectric Loss Model. J. Electron. Mater..

[20] Iglesias T.P., Vilão G., Reis J.C.R. (2017). An approach to the interpretation of Cole–Davidson and Cole–Cole dielectric functions. J. Appl. Phys..

[21] Wu Y., Han M., Liu T., Deng L. (2015). Studies on the microwave permittivity and electromagnetic wave absorption properties of Fe-based nano-composite flakes in different sizes. J. Appl. Phys..

[22] Wei Z., Li Z., Chen D., Liang J., Kong J. (2024). Recent Progress of Advanced Composites for Broadband Electromagnetic Wave Absorption. Small Struct..

[23] Zhang W., Dai F.-Z., Xiang H., Zhao B., Wang X., Ni N., Karre R., Wu S., Zhou Y. (2021). Enabling highly efficient and broadband electromagnetic wave absorption by tuning impedance match in high-entropy transition metal diborides (HE TMB2). J. Adv. Ceram..

[24] Zheng Y., Wu M., Qian C., Jin Y., Xiao W., Liang X. (2023). Tunable Electromagnetic and Microwave Absorption Properties of Magnetic FeNi_3_ Alloys. Nanomaterials.

[25] Li H., Li H., Yang F., Cai Q., Xu W., Wang R., Liu Y. (2024). Synthesis and Study of Electromagnetic Wave Absorption Performance of Nano Medium-Entropy FeCoNi Magnetic Alloy Particles with Varying Ni Element Contents. Met. Mater. Int..

[26] Li H., Li H., Yang F., Cai Q., Xu W., Wang R., Liu Y. (2024). Synthesis of nano/micro ternary magnetic particles with different Co content and their electromagnetic absorbing properties. J. Alloys Compd..

[27] Zhao H., Zhu Z., Xiong C., Zheng X., Lin Q. (2016). The influence of different Ni contents on the radar absorbing properties of FeNi nano powders. RSC Adv..

[28] Cheng Y., Ji G., Li Z., Lv H., Liu W., Zhao Y., Cao J., Du Y. (2017). Facile synthesis of FeCo alloys with excellent microwave absorption in the whole Ku-band: Effect of Fe/Co atomic ratio. J. Alloys Compd..

[29] Zhou T., Deng L., Liang D. (2008). Effect of Si content on ordering degree and electromagnetic characteristics in FeSiAl alloys. Acta Met. Sin..

[30] Feng J., Li Z., Jia Y., Yang B., Liu S., Zhao X., Li L., Zuo L. (2018). Significant high-frequency electromagnetic wave absorption performance of Ni_2+x_Mn_1−x_Ga alloys. J. Mater. Sci..

[31] Yang P., Liu Y., Zhao X., Cheng J., Li H. (2016). Electromagnetic wave absorption properties of mechanically alloyed FeCoNiCrAl high entropy alloy powders. Adv. Powder Technol..

[32] Duan Y., Li M., Guo Y., Zhu N., Pang H., Dou C. (2024). Improving electromagnetic wave absorption performance by adjusting the proportion of brittle BCC phase in FeCoNiCr0.4Mnx high-entropy alloys. Mater. Today Phys..

[33] Qiu Z., Liu X., Yang T., Wang J., Wang Y., Ma W., Huang Y. (2024). Synergistic Enhancement of Electromagnetic Wave Absorption and Corrosion Resistance Properties of High Entropy Alloy Through Lattice Distortion Engineering. Adv. Funct. Mater..

[34] Li Q., Zhang X., Chen Z., Li W., Ma H., Chen Y., Guan J. (2024). Improving interfacial magnetoelastic effect and complex permeability of FeSiAl alloy powders for broadband decimeter-wave absorption via Cr doping. J. Alloys Compd..

[35] Liu X., Sun H., Yan W., Yang S., Jiang X. (2023). Effect of Gd addition on the microwave absorption properties of FeSiAl composite. Vacuum.

[36] Tang J., Zhong L., Yang R., Yan H., Song T., Zhou T., Jiang L. (2022). Optimizing low frequency electromagnetic parameters by controlling the distribution of boron in FeSiAl alloy. J. Magn. Magn. Mater..

[37] Liu J., Ma T., Tong H., Luo W., Yan M. (2010). Electromagnetic wave absorption properties of flaky Fe–Ti–Si–Al nanocrystalline composites. J. Magn. Magn. Mater..

[38] Li Z., Wang J., Zhao F. (2021). Study on the electromagnetic properties and microwave absorbing mechanism of flaky FeSiAl alloy based on annealing and phosphate coating. Mater. Res. Express.

[39] Chen X., Zheng Z., Yu B., Xiao B., Qiu Z., Zeng D. (2025). The enhanced soft magnetic properties of FeSiBPCu nanocrystalline alloys by doping C and segmented annealing. J. Alloys Compd..

[40] Zou B., Zhou T., Hu J. (2013). Effect of amorphous evolution on structure and absorption properties of FeSiCr alloy powders. J. Magn. Magn. Mater..

[41] Feng Y., Qiu T. (2012). Enhancement of electromagnetic and microwave absorbing properties of gas atomized Fe-50wt%Ni alloy by shape modification. J. Magn. Magn. Mater..

[42] Liu J., Feng Y., Qiu T. (2011). Synthesis, characterization, and microwave absorption properties of Fe–40wt%Ni alloy prepared by mechanical alloying and annealing. J. Magn. Magn. Mater..

[43] Li Y., Zhang Y., Zhang C., Guan Z., Jiang J., Zhen L. (2024). Improve the electromagnetic wave absorbing properties of FeSiAl particles in the P/L/S bands by optimizing the ball milling process. Mater. Today Commun..

[44] Dong Y., Liang X., Zhong S., Yu M., Wang C. (2023). A simple method for preparing flaky FeSiAl for low-frequency electromagnetic wave absorber. J. Magn. Magn. Mater..

[45] Cai X.-D., Jiang X.-J., Xie W., Mu J.-Y., Yin D.-F. (2018). Effect of particle size on the preparation and microwave absorption properties of FeSiAl magnetically soft alloy hollow microspheres. Def. Technol..

[46] Zhu Y., Su Y., Lu Q., Li J. (2024). FeNi alloys with controllable composition for tunable magnetic properties and broadband absorption. Mater. Today Commun..

[47] Li H., Cao Z., Lin J., Zhao H., Jiang Q., Jiang Z., Liao H., Kuang Q., Xie Z. (2017). Synthesis of u-channelled spherical Fe_x_(Co_y_Ni_1−y_)_100−x_ Janus colloidal particles with excellent electromagnetic wave absorption performance. Nanoscale.

[48] Hang T., Zheng J., Zheng Y., Jiang S., Zhou L., Sun Z., Li X., Tong G., Chen Y. (2023). Wheat-like Ni-coated core–shell silver nanowires for effective electromagnetic wave absorption. J. Colloid Interface Sci..

[49] Yang P., Zhao X., Liu Y., Lai X. (2017). Preparation and electromagnetic wave absorption properties of hollow Co, Fe@air@Co and Fe@Co nanoparticles. Adv. Powder Technol..

[50] Li S., Huang Y., Ling D., Zhang N., Zong M., Qin X., Liu P. (2019). Enhanced microwave-absorption with carbon-encapsulated Fe-Co particles on reduced graphene oxide nanosheets with nanoscale-holes in the basal plane. J. Colloid Interface Sci..

[51] Somo T.R., Maponya T.C., Davids M.W., Hato M.J., Lototskyy M.V., Modibane K.D. (2020). A Comprehensive Review on Hydrogen Absorption Behaviour of Metal Alloys Prepared through Mechanical Alloying. Metals.

[52] Rangel M.M., Ramírez J.M.M., Mejía L.T., Barreto M.H.M., Muñoz B.C. (2021). Process control agent effect on the structural and magnetic properties of mechanically alloyed Fe(Al) disordered system. J. Magn. Magn. Mater..

[53] Xiong J., Pan S., Cheng L., Liu X., Lin P. (2015). Structure and microwave absorption properties of Pr–Fe–Ni alloys. J. Magn. Magn. Mater..

[54] Ajia S., Asa H., Toyoda Y., Sato M., Matsuura M., Tezuka N., Sugimoto S. (2022). Development of an alternative approach for electromagnetic wave absorbers using Fe–Cr–Co alloy powders. J. Alloys Compd..

[55] Jin M., Zhao F., Liu M. (2025). Enhanced permeability and reduced loss in FeSiAl soft magnetic composites via phosphating and ferrite coating. J. Alloys Compd..

[56] Zare Y., Shams M.H., Jazirehpour M. (2017). Tuning microwave permittivity coefficients for enhancing electromagnetic wave absorption properties of FeCo alloy particles by means of sodium stearate surfactant. J. Alloys Compd..

[57] Chu X., Lin S., Li H., Xu J., Li Z., Shu L., Pang M., Zhang H., Liu D. (2024). Heterointerface regulation of core-shell FeSiAl magnetic powders by in situ oxidation as high-efficiency absorbers. Ceram. Int..

[58] Aslam M.A., Ahsen R., Uddin W., Rehman S.U., Khan M.S., Bilal M., Li N., Wang Z. (2022). Tailoring the morphology of CoNi alloy by static magnetic field for electromagnetic wave absorption. Eur. Phys. J. Plus.

[59] Zhao H., Zhu Z., Xiong C., Xu X., Lin Q. (2017). The effect of transverse magnetic field treatment on wave-absorbing properties of FeNi alloy powders. J. Magn. Magn. Mater..

[60] Tao Z., Gu W., Tan X., Zhang H., Wu C., Xia A., Ji G. (2025). Carbon Materials: A Crucial Component Within the Family of High-Performance Microwave Absorbing Materials. Adv. Funct. Mater..

[61] Yan Z., Hao Z., Yajuan X., Yuexin D. (2010). Studies of Electromagnetic Properties of MWCNTs after Electroless Plating with Co-Fe Alloy. Chin. J. Aeronaut..

[62] Zhao H., Han X., Han M., Zhang L., Xu P. (2010). Preparation and electromagnetic properties of multiwalled carbon nanotubes/Ni composites by γ-irradiation technique. Mater. Sci. Eng. B.

[63] Zhao P.-Y., Wang H.-Y., Wang G.-S. (2020). Enhanced Electromagnetic Absorption Properties of Commercial Ni/MWCNTs Composites by Adjusting Dielectric Properties. Front. Chem..

[64] Huang L., Liu X., Chuai D., Chen Y., Yu R. (2016). Flaky FeSiAl alloy-carbon nanotube composite with tunable electromagnetic properties for microwave absorption. Sci. Rep..

[65] Liu Y., Qiang C. (2015). Magnetic properties and microwave absorption properties of short carbon fibres coated by Ni–Fe alloy coatings. Bull. Mater. Sci..

[66] Xiang J., Li J., Zhang X., Ye Q., Xu J., Shen X. (2014). Magnetic carbon nanofibers containing uniformly dispersed Fe/Co/Ni nanoparticles as stable and high-performance electromagnetic wave absorbers. J. Mater. Chem. A.

[67] Zuo X., Xu P., Zhang C., Li M., Jiang X., Yue X. (2019). Porous magnetic carbon nanofibers (P-CNF/Fe) for low-frequency electromagnetic wave absorption synthesized by electrospinning. Ceram. Int..

[68] Wang L., He F., Wan Y. (2011). Facile synthesis and electromagnetic wave absorption properties of magnetic carbon fiber coated with Fe–Co alloy by electroplating. J. Alloys Compd..

[69] Yang B., Fang J., Xu C., Cao H., Zhang R., Zhao B., Huang M., Wang X., Lv H., Che R. (2022). One-Dimensional Magnetic FeCoNi Alloy Toward Low-Frequency Electromagnetic Wave Absorption. Nano-Micro Lett..

[70] Cai W., Jiang J., Zhang Z., Liu Z., Zhang L., Long Z., Bi K. (2024). Carbon nanofibers embedded with Fe–Co alloy nanoparticles via electrospinning as lightweight high-performance electromagnetic wave absorbers. Rare Met..

[71] Wang B., Ding M., Shao C., Yu J., Kong H., Zhao D., Li C. (2023). Facile synthesis of CoxFey@C nanocomposite fibers derived from pyrolysis of cobalt/iron chelate nanowires for strong broadband electromagnetic wave absorption. Chem. Eng. J..

[72] Chen Y., Lei Z., Wu H., Zhu C., Gao P., Ouyang Q., Qi L.-H., Qin W. (2013). Electromagnetic absorption properties of graphene/Fe nanocomposites. Mater. Res. Bull..

[73] Zhao S., Wang C., Zhong B. (2020). Optimization of electromagnetic wave absorbing properties for Ni-Co-P/GNs by controlling the content ratio of Ni to Co. J. Magn. Magn. Mater..

[74] Xie H., Li J., Yang R., Yang J., Wang T., Wang Q. (2024). Controllable fabrication of CoNi bimetallic alloy for high-performance electromagnetic wave absorption. RSC Adv..

[75] Liang X., Wang C., Yao Z., Zhang Y., Liu S., Liu J., Yu M. (2022). A facile synthesis of Fe/C composite derived from Fe-metal organic frameworks: Electromagnetic wave absorption with thin thickness. J. Alloys Compd..

[76] Zhang R., Hu Q., Yang S., Yan S., Gu Y., Ji J., Zhou Z., Liu C., Li X., Wang Z. (2024). MOF-Fe@C Nanocomposites for Microwave Absorption. Adv. Eng. Mater..

[77] Huang M., Wang L., Pei K., You W., Yu X., Wu Z., Che R. (2020). Multidimension-Controllable Synthesis of MOF-Derived Co@N-Doped Carbon Composite with Magnetic-Dielectric Synergy toward Strong Microwave Absorption. Small.

[78] Li L., Li G., Ouyang W., Zhang Y., Zeng F., Liu C., Lin Z. (2021). Bimetallic MOFs derived FeM(II)-alloy@C composites with high-performance electromagnetic wave absorption. Chem. Eng. J..

[79] Xiong J., Xiang Z., Zhao J., Yu L., Cui E., Deng B., Liu Z., Liu R., Lu W. (2019). Layered NiCo alloy nanoparticles/nanoporous carbon composites derived from bimetallic MOFs with enhanced electromagnetic wave absorption performance. Carbon.

[80] Zhang L., Zhang W., Rehman S.U., Shen S., Liu Y., Long F., Du H., Dong W., Hu Y., Zou H. (2023). Optimization of microwave absorption properties of flaky FeSiAl magnetic alloy by surface modification. J. Alloys Compd..

[81] Chang Q., Li C., Sui J., Waterhouse G.I., Zhang Z.-M., Yu L.-M. (2022). Ni/Ni_3_ZnC_0.7_ modified alginate-derived carbon composites with porous structures for electromagnetic wave absorption. Carbon.

[82] Yang X., Wang H., Chen J., An Q., Xiao Z., Hao J., Zhai S., Sheng J. (2024). Customization of FeNi alloy nanosheet arrays inserted with biomass-derived carbon templates for boosted electromagnetic wave absorption. Int. J. Miner. Met. Mater..

[83] Su Z., Yi S., Zhang W., Tian L., Zhang Y., Zhou S., Niu B., Long D. (2022). Magnetic-Dielectric Complementary Fe-Co-Ni Alloy/Carbon Composites for High-Attenuation C-Band Microwave Absorption via Carbothermal Reduction of Solid-Solution Precursor. Adv. Electron. Mater..

[84] Lyu L., Wang F., Zhang X., Qiao J., Liu C., Liu J. (2021). CuNi alloy/ carbon foam nanohybrids as high-performance electromagnetic wave absorbers. Carbon.

[85] Zou Y., Wang L., Fan B., Yue J., Liu Y., Huang X. (2025). (FeNi)x(SiO2)1-x nano-granular film modified carbon foam for broad-band microwave absorption. Appl. Surf. Sci..

[86] Zou Y., Huang X., Fan B., Yue J., Liu Y. (2022). Enhanced low-frequency microwave absorption performance of FeNi alloy coated carbon foam assisted by SiO_2_ layer. Appl. Surf. Sci..

[87] Gao Y., Chen X., Jin X., Zhang C., Zhang X., Liu X., Li Y., Li Y., Lin J., Gao H. (2024). Multifunction integration within magnetic CNT-bridged MXene/CoNi based phase change materials. eScience.

[88] Li X., Wen C., Yang L., Zhang R., Li X., Li Y., Che R. (2021). MXene/FeCo films with distinct and tunable electromagnetic wave absorption by morphology control and magnetic anisotropy. Carbon.

[89] He J., Liu X., Deng Y., Peng Y., Deng L., Luo H., Cheng C., Yan S. (2021). Improved magnetic loss and impedance matching of the FeNi-decorated Ti_3_C_2_T MXene composite toward the broadband microwave absorption performance. J. Alloys Compd..

[90] Lei C., Ge C., Ge X., Qian J., Du Y. (2022). Enhanced microwave absorption of flaky FeSiAl/ZnO composites fabricated via precipitation. Mater. Sci. Eng. B.

[91] Wang C., Wang H., Wang B., Ye T., Xue J., Wei H. (2024). A ZrO2-wrapped FeSiAl composite with a cotton-like structure for broadband electromagnetic wave absorption. Mater. Res. Bull..

[92] Xiang H., Liu Y., Liu Y., Su E., Su X. (2025). Electromagnetic wave absorbing properties of flexible thermal conductive ZnO@FeSiAl silicone rubber. Mater. Today Nano.

[93] Liu J., Liu C., Tong Y., Sun H., Peng H., Zhang M., Wu S., Zhang H., Gong H., Zheng Z. (2022). Enhanced EMW absorption properties of SiCN/Fe/Ni ceramics modified with Fe/Ni bimetal. Ceram. Int..

[94] Javid M., Qu X., Huang F., Li X., Farid A., Shah A., Duan Y., Zhang Z., Dong X., Pan L. (2021). In-situ synthesis of SiC/Fe nanowires coated with thin amorphous carbon layers for excellent electromagnetic wave absorption in GHz range. Carbon.

[95] Wang H., Ma N., Yan Z., Deng L., He J., Hou Y., Jiang Y., Yu G. (2015). Cobalt/polypyrrole nanocomposites with controllable electromagnetic properties. Nanoscale.

[96] Almasi-Kashi M., Mokarian M.H., Alikhanzadeh-Arani S. (2018). Improvement of the microwave absorption properties in FeNi/PANI nanocomposites fabricated with different structures. J. Alloys Compd..

[97] Yang Y., Zhu Y. (2023). Preparation and microwave absorbing properties of core shell Fe/Fe 3C@carbon@polyaniline multiphase microwave absorbing materials. J. Zhejiang Sci-Tech Univ..

[98] Li X., Cui E., Xiang Z., Yu L., Xiong J., Pan F., Lu W. (2020). Fe@NPC@CF nanocomposites derived from Fe-MOFs/biomass cotton for lightweight and high-performance electromagnetic wave absorption applications. J. Alloys Compd..

[99] Kong M., Liu X., Jia Z., Wang B., Wu X., Wu G. (2021). Porous magnetic carbon CoFe alloys@ZnO@C composites based on Zn/Co-based bimetallic MOF with efficient electromagnetic wave absorption. J. Colloid Interface Sci..

[100] Zhou L., Yu J., Wang Z., Wang H., Huang J., Mu W., Zheng H. (2020). Electromagnetic and microwave absorption properties of FeSiAl and flaky graphite filled Al_2_O_3_ composites with different FeSiAl particle size. Ceram. Int..

[101] Liu N., Dou Y., Zhang X., Yu L., Yan X. (2022). Design of porous FeNi-carbon nanosheets by a double-effect synergistic strategy for electromagnetic wave absorption. Carbon.

[102] Li C., Chen G., Jiang W., Jiang X., Yan X. (2021). High-performance electromagnetic wave absorption of FeNi/N, S-codoped carbon composites in 2–40 GHz. Carbon.

[103] Koyappayil A., Yagati A.K., Lee M.-H. (2023). Recent Trends in Metal Nanoparticles Decorated 2D Materials for Electrochemical Biomarker Detection. Biosensors.

[104] Li T., Zhi D.-D., Guo Z.-H., Li J.-Z., Chen Y., Meng F.-B. (2021). 3D porous biomass-derived carbon materials: Biomass sources, controllable transformation and microwave absorption application. Green Chem..

[105] Liu Y., He J., Zhang N., Zhang W., Zhou Y., Huang K. (2021). Advances of microwave plasma-enhanced chemical vapor deposition in fabrication of carbon nanotubes: A review. J. Mater. Sci..

[106] Wang H., Dai Y.Y., Geng D.Y., Ma S., Li D., An J., He J., Liu W., Zhang Z.D. (2015). Co_x_Ni_100−x_ nanoparticles encapsulated by curved graphite layers: Controlled in situ metal-catalytic preparation and broadband microwave absorption. Nanoscale.

[107] Prokhorenkova N., Zhilkashinova A., Abilev M., Łatka L., Ocheredko I., Zhilkashinova A. (2025). Phase Composition, Structure, and Microwave Absorption of Magnetron-Sputtered Co–C–Cr Multilayer Films. Compounds.

[108] Liu D., Zhang Y., Zhou C., Lv H., Chen S., Chen Y., Gao S., Zhang B. (2020). A facile strategy for the core-shell FeSiAl composites with high-efficiency electromagnetic wave absorption. J. Alloys Compd..

[109] Saini L., Gupta V., Patra M.K., Jani R.K., Shukla A., Kumar N., Dixit A. (2021). Impedance engineered microwave absorption properties of Fe-Ni/C core-shell enabled rubber composites for X-band stealth applications. J. Alloys Compd..

[110] Xie Z., Geng D., Liu X., Ma S., Zhang Z. (2011). Magnetic and Microwave-absorption Properties of Graphite-coated (Fe, Ni) Nanocapsules. J. Mater. Sci. Technol..

[111] Wang Y., Wang W., Sun J., Sun C., Feng Y., Li Z. (2018). Microwave-based preparation and characterization of Fe-cored carbon nanocapsules with novel stability and super electromagnetic wave absorption performance. Carbon.

[112] Liu X., Li B., Geng D., Cui W., Yang F., Xie Z., Kang D., Zhang Z. (2009). (Fe, Ni)/C nanocapsules for electromagnetic-wave-absorber in the whole Ku-band. Carbon.

[113] Chen N., Dong Z., Wang X.-Y., Guan Z.-J., Jiang J.-T., Wang K.-J. (2022). Construction of FeNi_3_ and core–shell structured FeNi_3_@C microspheres toward broadband electromagnetic wave absorbing. Appl. Surf. Sci..

[114] Liu Q., Dai J., Hu F., Zhang Z., Xiong K., Xu G. (2021). Core-shell structured Fe/ZnO composite with superior electromagnetic wave absorption performance. Ceram. Int..

[115] Wang B., Chen H., Wang S., Shi Y., Liu X., Fu Y., Liu T. (2021). Construction of core-shell structured Co_7_Fe_3_@C nanocapsules with strong wideband microwave absorption at ultra-thin thickness. Carbon.

[116] Chen G., Zhang R., Yuan M., Xue S., Liu Y., Li B., Luo K., Lai Y., Zhang J., Lv H. (2024). Visualizing Nanoscale Interlayer Magnetic Interactions and Unconventional Low-Frequency Behaviors in Ferromagnetic Multishelled Structures. Adv. Mater..

[117] Liu X., Ou Z., Geng D., Han Z., Wang H., Li B., Brück E., Zhang Z. (2010). Enhanced absorption bandwidth in carbon-coated supermalloy FeNiMo nanocapsules for a thin absorb thickness. J. Alloys Compd..

[118] Zhao B., Du Y., Lv H., Yan Z., Jian H., Chen G., Wu Y., Fan B., Zhang J., Wu L. (2023). Liquid-Metal-Assisted Programmed Galvanic Engineering of Core–shell Nanohybrids for Microwave Absorption. Adv. Funct. Mater..

[119] Qu X., Javid M., Li X., Huang F., Li P., Duan Y., Zhang Z., Zhang X., Dong X. (2023). Tailoring of electromagnetic properties of arc-produced carbon-coated FeNi alloy nanocapsules for highly efficient microwave absorption. Diam. Relat. Mater..

[120] Mu S., Wang S., Pang X., Pu J. (2023). Investigation of electromagnetic wave absorption and anti-corrosion mechanism of multifunctional Ni/AlN composite coatings with core-shell structure by laser cladding. J. Alloys Compd..

[121] Zhang X., Guo Y., Ali R., Tian W., Liu Y., Zhang L., Wang X., Zhang L., Yin L., Su H. (2020). Bifunctional carbon-encapsulated FeSiAl hybrid flakes for enhanced microwave absorption properties and analysis of corrosion resistance. J. Alloys Compd..

[122] Yan X., Liu F., Guo J., Deng Y., Yu C., Lv S., Jiang X. (2023). Excellent electrically insulated, thermal-oxidation resistant and corrosion resistant flaky-FeSiAl/BN core–shell composites for high-performance microwave absorption in harsh environments. Compos. Part A Appl. Sci. Manuf..

[123] Liu Q., Cao Q., Bi H., Liang C., Yuan K., She W., Yang Y., Che R. (2015). CoNi@SiO_2_@TiO_2_ and CoNi@Air@TiO_2_ Microspheres with Strong Wideband Microwave Absorption. Adv. Mater..

[124] Wu J., Wang K., Wang K., Park H., Hwang J., Li J., Wang Y., Hu J., Xiong S. (2025). Coral-Like Yolk–Shell-Structured CoNi@Void@C Microspheres for Enhanced Microwave Absorption, Photothermal, Anti-Corrosion, and Radiation Shielding Properties. Rare Met..

[125] Wu H., Lan D., Li B., Zhang L., Fu Y., Zhang Y., Xing H. (2019). High-entropy alloy@air@Ni–NiO core-shell microspheres for electromagnetic absorption applications. Compos. Part B Eng..

[126] Wang B., Li C., Ni C., Xie X. (2022). Facile synthesis of heterogeneous Co/MnO@C nanocapsules with dual-cores: Achieving strong and broadband microwave absorption by magnetic-dielectric synergy. Appl. Surf. Sci..

[127] Hou Y., Li B., Chen J., Shen X., Wang B., Liu K., Wei S., He X., Li D., Han Q. (2023). Electromagnetic wave absorption properties of core double-shell structured α-Fe(Si)@Fe_3_O_4_@SiO_2_ composites. Appl. Surf. Sci..

[128] Shi G., Zhang B., Wang X., Fu Y. (2016). Enhanced microwave absorption properties of core double-shell type Fe@C@BaTiO_3_ nanocapsules. J. Alloys Compd..

[129] Zhang H., Cao F., Xu H., Tian W., Pan Y., Mahmood N., Jian X. (2022). Plasma-enhanced interfacial engineering of FeSiAl@PUA@SiO_2_ hybrid for efficient microwave absorption and anti-corrosion. Nano Res..

[130] Huang B., Hu H., Lim S., Tang X.-Z., Huang X., Liu Y., Yue J. (2022). Gradient FeNi-SiO_2_ films on SiC fiber for enhanced microwave absorption performance. J. Alloys Compd..

[131] Zhou Y., Qian J., Qiang Z., Kong W., Ye C., Zhu M. (2024). Interface engineering of Fe/Fe_3_C@C magnetic-carbon composites for superior microwave absorption. Compos. Part A Appl. Sci. Manuf..

[132] Wang H., Chen W., Hang T., Li Z., Wang X., Chen Y., Zheng J. (2024). Multi-scale heterogeneous composite elastomer absorbers synergistically enhanced by CoNi nanospheres and carbon nanotubes. J. Colloid Interface Sci..

[133] Guan X., Jiang M., Shen Z., Tan S., Ji G., Xu Z.J. (2025). High-Entropy Interface Engineering in Multifunctional Green Fiber Aerogels for Coupled Electromagnetic and Waste-Heat Management. Adv. Funct. Mater..

[134] Cheng Z., Zhou J., Liu Y., Yan J., Wang S., Tao J., Zou K., Tan R., Yao Z. (2023). 3D printed composites based on the magnetoelectric coupling of Fe/FeCo@C with multiple heterogeneous interfaces for enhanced microwave absorption. Chem. Eng. J..

[135] Shu Y., Zhao T., Li Y., Yang L., Li X., Feng G., Jia W., Luo F. (2023). Porous Fe/FeO/Fe_2_O_3_ nanorod/RGO composites with high-efficiency electromagnetic wave absorption property. Appl. Surf. Sci..

[136] Ma S., Jing T., Wan Y., Shen F., Liu X. (2025). Constructing metal/metal heterointerfaces in lamellar structured FeSiCr@Fe_3_O_4_ composites for stable, strong and broad microwave absorption via FeSiAl nanoparticles embedding. Appl. Surf. Sci..

[137] Zhou H., Lin Y., Ma Y., Han L., Cai Z., Cheng Y., Yuan Q., Huang W., Yang H., Che R. (2024). Hierarchical structure Fe@CNFs@Co/C elastic aerogels with intelligent electromagnetic wave absorption. InfoMat.

[138] Li X., Qu X., Xu Z., Dong W., Wang F., Guo W., Wang H., Du Y. (2019). Fabrication of Three-Dimensional Flower-like Heterogeneous Fe_3_O_4_/Fe Particles with Tunable Chemical Composition and Microwave Absorption Performance. ACS Appl. Mater. Interfaces.

[139] Li X., Du D., Wang C., Wang H., Xu Z. (2017). In situ synthesis of hierarchical rose-like porous Fe@C with enhanced electromagnetic wave absorption. J. Mater. Chem. C.

[140] Shu Y., Zhao T., Li X., Yang L., Cao S., Ahmad A., Jiang T., Luo H., Jing Z., Ain N.U. (2022). Flower-like Co@CoO nanohybrids assembled by crisp-rice-like quadrate flakes as high-performance electromagnetic wave absorber. Appl. Surf. Sci..

[141] Li C., Sui J., Jiang X., Zhang Z., Yu L. (2020). Efficient broadband electromagnetic wave absorption of flower-like nickel/carbon composites in 2–40 GHz. Chem. Eng. J..

[142] Bao S., Zhang M., Bu X., Zhang W., Jiang Z., Xie Z. (2023). Combinatorial Structural Engineering of Multichannel Hierarchical Hollow Microspheres Assembled from Centripetal Fe/C Nanosheets to Achieve Effective Integration of Sound Absorption and Microwave Absorption. ACS Appl. Mater. Interfaces.

[143] Cao S., Peng L., Han T., Liu B., Zhu D., Zhao C., Xu J., Tang Y., Wang J., He S. (2020). Hydrothermal synthesis of nanoparticles-assembled NiO microspheres and their sensing properties. Phys. E Low-Dimens. Syst. Nanostructures.

[144] Wang L., Wen B., Yang H., Qiu Y., He N. (2020). Hierarchical nest-like structure of Co/Fe MOF derived CoFe@C composite as wide-bandwidth microwave absorber. Compos. Part A Appl. Sci. Manuf..

[145] Xu C., Wang L., Li X., Qian X., Wu Z., You W., Pei K., Qin G., Zeng Q., Yang Z. (2021). Hierarchical Magnetic Network Constructed by CoFe Nanoparticles Suspended Within “Tubes on Rods” Matrix Toward Enhanced Microwave Absorption. Nano-Micro Lett..

[146] Liang X., Man Z., Quan B., Zheng J., Gu W., Zhang Z., Ji G. (2020). Environment-Stable CoxNiy Encapsulation in Stacked Porous Carbon Nanosheets for Enhanced Microwave Absorption. Nano-Micro Lett..

[147] Deng J., Zhang X., Zhao B., Bai Z., Wen S., Li S., Li S., Yang J., Zhang R. (2018). Fluffy microrods to heighten the microwave absorption properties through tuning the electronic state of Co/CoO. J. Mater. Chem. C.

[148] Sun X., Li W., Qu H., Wang T., Han R., Feng H., Wu W., Shui J., He J., Wang T. (2024). Multi-scale structural design of multilayer magnetic composite materials for ultra-wideband microwave absorption. Carbon.

[149] He L., Li X., Zhao Y., Zhong Z., Zhang J., Yang Y., Xi X. (2022). The multilayer structure design of magnetic-carbon composite for ultra-broadband microwave absorption via PSO algorithm. J. Alloys Compd..

[150] Zou Y., Huang X., Fan B., Liu Y., Yue J. (2023). Triple-layer structure of carbon foam coated with carbon nanotubes and FeNi alloy for high-performance electromagnetic wave absorption. J. Alloys Compd..

[151] Qu N., Sun H., Sun Y., He M., Xing R., Gu J., Kong J. (2024). 2D/2D coupled MOF/Fe composite metamaterials enable robust ultra–broadband microwave absorption. Nat. Commun..

[152] Fang D., Liu S., Li J., Jin H. (2023). Absorber design based on In/C@Co/C composites for efficient microwave absorption. J. Alloys Compd..

[153] Guo M., Wang Y., Liu S. (2024). Research on microwave absorption properties of FeCoGe composites and achieving ultra-broad bandwidth via metamaterial design. J. Mater. Sci. Mater. Electron..

[154] Liu C., Zhu L., Zheng S., Xu L., Yan L., Zhang Y., Ji G. (2025). A novel elastic dome array structure for precise pressure regulation toward tunable microwave absorption. Acta Mater..

[155] Feng M., Yu G., Zhang K., Li Y., Cheng H., Liang B. (2025). Electromagnetic-mechanical collaborative design of high-performance electromagnetic sandwich metastructure by machine learning based genetic optimization. J. Mater. Sci. Technol..

[156] Wang X., Guo M., Dai Y., Liang L., Tang D., Zhang B., Yang Y. (2023). Microwave All-Dielectric Metamaterial Design of FeSiAl/MWCNT Composite for Low-Frequency Broadband-Absorbing Properties. Metals.

